# Metabolic reprogramming of tomato roots during rhizobacteria-mediated defense against *Erwinia persicina*: modulation by gold nanoparticle conjugation

**DOI:** 10.3389/fpls.2026.1824882

**Published:** 2026-06-17

**Authors:** Nedaa Ali, Narjes Haje Dashti, Anandavalli Inbamani Raju

**Affiliations:** Department of Biological Sciences, Faculty of Science, Kuwait University, Sabah Al-Salem University City, Al-Shaddadiya, Kuwait

**Keywords:** defense metabolism, gold nanoparticles, plant-pathogen interactions, rhizobacteria -induced systemic resistance, rhizosphere metabolomics, *Solanum lycopersicum*

## Abstract

Rhizobacteria-induced systemic resistance (ISR) is an established strategy for enhancing plant tolerance to biotic stress, yet its metabolic consequences under nanoparticle-assisted delivery remain poorly understood. Here, we investigated metabolic reprogramming in tomato roots (*Solanum lycopersicum* L.) challenged with the pathogen *Erwinia persicina* following treatment with PGPR strain *Stenotrophomonas rhizophila* (Sr) applied either alone or conjugated to phycosynthesized gold nanoparticles using *Caulerpa sertularioides*. Bionanogold synthesis was confirmed by UV–Visible surface plasmon resonance (~534 nm). Successful conjugation with *S. rhizophila* (Sr-AuNPs) was validated via TEM, FTIR, dynamic light scattering (size increase from 78.15 ± 10.89 nm to 90.96 ± 1.96 nm), zeta potential (−28.56 mV), and ICP-MS, indicating stable nanoparticle–bacteria association. The integrated metabolic fingerprinting of tomato root exudates obtained from GC–MS, LC–MS/MS, and ^1^H NMR data was normalized and autoscaled prior to multivariate analysis. The variations in metabolic signatures associated with tomato roots under different treatments- control (T1), rhizobacteria (T2: Sr+Ep), rhizobacteria conjugated with nanoparticles (T3: Sr-AuNPs+Ep), and pathogens (T4: Ep) were characterized and distinguished by multivariate analysis. Various metabolites with distinct signatures were observed among the different treatments through one-way ANOVA test with FDR adjustment. These included LC–MS/MS m/z 338.33 (putative signature 13-docosenamide or Tentative lipid amide (C22), long chain lipid-associated ions (m/z 337.06), derivatives of Benzoic acid, oleanitrile and ^1^H NMR peaks related to lipid, Citrate/succinate, and oxygenated compounds. Pathway topology analysis revealed that the TCA cycle, Flavonoid biosynthesis, glyoxylate and dicarboxylate metabolism, and Cutin/Suberin/Wax biosynthesis were some of the more significant pathways represented in the detected metabolite data set. These pathway-level associations should be regarded as preliminary indications of functional relationships among the detected metabolites, rather than direct or conclusive evidence of pathway activation or metabolic flux changes. FTIR analysis further supported treatment-associated biochemical variation in root exudates. Collectively, the nanoparticle-conjugated rhizobacterial treatment was associated with a metabolite profile distinct from both the pathogen-only and rhizobacteria-only treatments. This provides a preliminary metabolomic framework for understanding nano-enabled plant–microbe interactions under biotic stress.

## 1 Introduction

The rapid rise of opportunistic pathogens affecting plants, as well as severe global climate change pressures and impacts from microbial ecosystems, has necessitated an unprecedented change in the way agricultural practices are managed. For example, the bacterial phytopathogen *Erwinia persicina* represents a threat to tomato production (*Solanum lycopersicum* L.). It can exist as both an epiphyte and endophyte, behaving like a harmless commensal until environmental triggers turn it virulent, causing wilt and fruit rot (Nechwatal and Theil, 2019; Wasendorf et al., 2022; Ning et al., 2025).

Additional concerns have arisen due to an increasing number of resistant strains emerging within populations of traditional bacterial phytopathogens and the ecological implications of utilizing conventional bactericidal methods to control bacterial phytopathogen populations.

In response, the integration of nanotechnology and microbiology, termed “nano-bio” intervention offers a promising frontier for precision agriculture. More precisely, bio-nanocomposites provide a multi-functional platform for both pathogen suppression and plant growth promotion (Dutta et al., 2023; Masood et al., 2024). Although many studies have focused on chemically synthesized nanoparticles, there is an emerging mandate for “green” synthesis pathways (Dauthal and Mukhopadhya, 2016; Mukherjee et al., 2021; Sidorowicz et al., 2023). Phycosynthesis, a methodology based on the use of extracts from marine macroalgae for the reduction and stability of metal ions to nanoparticles, is an eco-friendly method. Marine macroalgae such as *Caulerpa* sp. Are rich in bioactive compounds such as sulfated polysaccharides, polyphenols, and pigments, which are not only efficient reducers but are also powerful immunity elicitors of plants (Yasman and Akhmad, 2025).

Besides the physical delivery of the nanoparticles, the importance of the plant-microbiome interface in plant wellness is of primary importance. Stress Protecting Agents (SPAs), such as the rhizobacterium *Stenotrophomonas rhizophila* (Sr), play a critical role in the stabilization of the root microbe complex through the microbe’s ability to generate signaling and osmoprotector molecules (Alavi et al., 2013; Ulrich et al., 2021; Gupta et al., 2022; Dunn and Becerra-Rivera, 2023). Previous studies through genomics and metabolomics have shown the ability of *S. rhizophila* to prime the plant for a state of systemic acquired resistance (SAR), thus placing the plant in a protective state from pathogenic attack (Berg et al., 2002; Adeleke et al., 2021; Kumar et al., 2023; Raio et al., 2023). With the inclusion of phycosynthesized gold nanoparticles (AuNPs), it has been postulated that a synergistic interaction takes place, such that the gold nanoparticles can be used as “amplified priming agents.”

The synergy between the *S. rhizophila* PGPR strain and the AuNPs is reflected in the dual benefits they confer to the rhizosphere. On the microbial side, AuNPs stimulate bacterial attachment, biofilm formation, and stable root localization. This microenvironment enhancement results in the increased production of microbial molecules that induce systemic resistance (ISR), including phytohormones, siderophores, and secondary elicitors (Pajerski et al., 2019; Panichikkal et al., 2019; Zhu et al., 2023; Thakur et al., 2025). On the plant side, the nanoparticles act as internal primers. They directly modulate key intracellular processes, including reactive oxygen species (ROS) signaling dynamics and antioxidant enzyme activity. These modifications are primarily coordinated through the activation of the salicylic acid and jasmonic acid defense pathways (Khan et al., 2022; Polyakov et al., 2023).

Recent reports have shown that nanoparticle-induced priming boosts plant immunity by regulating physiology, transcription, and metabolism, without compromising overall fitness costs in comparison with defense response induction (Mahakham et al., 2017; Venzhik et al., 2025; Tripathi et al., 2022; Yadav et al., 2024). Likewise, PGPR has been recognized to influence plant immunity through manipulation of signal transduction and gene expression related to defense pathways (Dunn and Becerra-Rivera, 2023; Dutta et al., 2023). Hence, the synergistic interaction between AuNPs and PGPR enhances ISR by improving microbial functions and enhancing the delivery of active signals as well as manipulating host defense responses (Verma et al., 2024).

Nevertheless, the induction of such high-level defense mechanisms is not a “free “biological process. Plants operate under finite resource constraints, giving rise to the concept of “Metabolic Drag”. Metabolic drag is used as an operational term describing the reduction in relative abundance of primary metabolite signals accompanying increased allocation toward defense-related metabolism (Zrimec et al., 2025; Zaman et al., 2026). According to this theory, energetic resources directed towards defense signaling hubs, including Flavonoid biosynthesis and the production of protective secondary metabolites, are often reallocated away from primary metabolic pathways. Consequently, while treatments that enhance defense responses can successfully mitigate pathogen stress, the diversion of energy flux away from structural biomass synthesis such as cell wall polysaccharides and pectin esters may adversely affect overall plant vigor (Xiao and Anderson, 2013; Huot et al., 2014; Di Silvestre et al., 2021; Pan et al., 2025).

Modern systems biology tries to unravel these complex tripartite interactions, host-pathogen-nanocomposite, by using multi-platform metabolomics (Akhtar et al., 2020; Wang et al., 2024; Ning et al., 2025). Combining information from liquid chromatography-mass spectrometry (LC-MS/MS), gas chromatography-mass spectrometry (GC-MS), and proton nuclear magnetic resonance (^1^H NMR), a comprehensive “snapshot” can be captured about the physiological state of a plant (Patel et al., 2021). GC-MS, LC-MS/MS, and ^1^H NMR spectroscopy allows for complementarity in metabolome coverage, thus improving the identification and quantification of metabolites and their associated pathways. The GC-MS technique is effective for volatile and derivatized primary metabolites. LC-MS/MS is highly sensitive towards semi-polar and secondary metabolites, and ^1^H NMR allows for reliable quantification of metabolites without the need for extensive sample pre-processing.

Despite the above strengths associated with these techniques, there are several challenges that exist, particularly regarding sensitivity, dynamic range, and coverage of metabolites (Lu et al., 2017). For example, low-abundance metabolites cannot be detected using ^1^H NMR, derivatization is required in GC-MS experiments, whereas GC-MS can only detect metabolites that are thermally stable, and LC-MS/MS data sets are affected by ionization efficiency and matrix effect issues.

In addition, the use of datasets from multiple platforms raises certain analytical issues, such as structural dissimilarities between data from different platforms, aligning of corresponding features, normalization procedures, and potential bias towards metabolites’ representations at each platform. Data preprocessing, normalization, and multivariate statistical methods will be necessary for solving these analytical problems.

In the present work, an integrated metabolomics approach based on several analytical platforms was utilized. As described in 2.9, integrated metabolomics involves complete data preprocessing and statistical analysis to minimize platform-dependent bias while also maximizing the unique strengths of the combined techniques (Pang et al., 2024).

Such tools permit the identification of specific biomarkers, including tentative lipid amide (C22), a lipid-derived defense mediator increasingly associated with pathogen- responsive lipid remodeling (Bach and Faure, 2010; De Bigault Du Granrut and Cacas, 2016; Wang et al., 2025). Benzoic acid is also identified as a well-established marker of microbially induced priming and systemic defense activation (Zeier, 2013; Yang et al., 2015; Zhang et al., 2025). The enrichment of Erucamide under phytopathogen infection supports its proposed role in stress-associated metabolic reprogramming. Meanwhile, Benzoic acid accumulation reflects activation of systemic resistance pathways linked to phenylpropanoid metabolism (Dixon and Paiva, 1995; Ikram et al., 2025).

Although ISR triggered by PGPR has been widely reported, its associated energy costs often result in a growth–defense trade-off (Pieterse et al., 2014; Pršić and Ongena, 2020). Nano-enabled elicitors have been proposed to mitigate this limitation (Noman et al., 2023; Lala, 2025); however, a critical gap remains in understanding how metal-based nanoparticles modulate plant–microbe interactions at the metabolic level to rebalance energy allocation between growth and defense (Panichikkal et al., 2019; Masood et al., 2024; Ning et al., 2025).

In particular, the metabolic branching points are key biochemical junctions that govern the partitioning of resources toward either growth-related processes or structural defense mechanisms such as Wax and Suberin biosynthesis. However, these points remain poorly characterized in nano-enabled ISR systems.

To address this gap, this study develops a novel phyco-conjugated *Stenotrophomonas rhizophila*–AuNP hybrid as a nano-enabled ISR platform. High-resolution SEM-EDX surface characterization, combined with multiplatform metabolomics (GC-MS, LC-MS/MS, ^1^H NMR and FTIR), is employed to elucidate how these chemical interactions between the bionanocomposite and the root surface modulate plant metabolic networks. This modulation helps optimize energy allocation during ISR against *Erwinia persicina* without compromising growth. This study employs pathway topology analysis to identify key metabolic associations, highlighting central hubs like the tricarboxylic acid (TCA) cycle that are associated with transitions between growth- and defense-related metabolic states. By linking nano-enabled ISR with metabolite-level reprogramming, this study provides mechanistic insight into how nanotechnology can alleviate the growth–defense trade-off. Ultimately, it establishes phyco-nanotechnology as a promising, non-toxic strategy for managing phytopathogenic bacteria while quantifying the associated metabolic costs.

## 2 Materials and methods

### 2.1 Preparation of Sr–AuNP conjugates

The phycosynthesized gold nanoparticles (AuNPs) obtained from the aqueous extract of *Caulerpa sertularioides* (2%, w/v) were treated with the log phase cultures of the PGPR or rhizobacterial strain of *Stenotrophomonas rhizophila* under a sterile environment. In brief, bacterial suspensions with OD_600_ ~0.1 were combined with AuNPs (100 µg/mL), followed by gentle stirring at 100 rpm for 20–30 minutes to promote conjugation via surface binding between the AuNPs and bacteria. After incubation, the Sr-AuNP conjugates were separated by centrifugation and washed thoroughly with sterile PBS solution.

### 2.2 Physicochemical characterization of nanoparticles

Hydrodynamic size and polydispersity index (PDI) values were analyzed through dynamic light scattering (DLS) The surface charge and colloidal stability properties were evaluated using zeta potentials analysis in Zetasizer Nano ZS (Malvern Instruments, UK). The FTIR spectrum of the samples was measured from 4000 to 400 cm^-^¹ to characterize functional groups that stabilize the nanoparticles and interact with bacteria (Jasco FT/IR-6300 spectrometer). Transmission electron microscope (TEM) (JEOL JEM-1400 Flash, Japan) and FESEM–EDS Field-emission scanning electron microscope JEOL JSM-7001F equipped with Oxford INCAx-act EDS detector (JEOL Ltd., Japan) were employed for morphological and structural characterization. Finally, the amount of gold was quantified in association with bacterial cells by Inductively coupled plasma mass spectrometer (ICP-MS, PerkinElmer NexION 2000P, US).

### 2.3 Plant material and growth conditions

Tomato (*Solanum lycopersicum* L. cv. GVS 51306 Super Marmande) seeds were surface-sterilized with 2% (v/v) sodium hypochlorite containing Tween-20 for 5 min, rinsed thoroughly with sterile distilled water, and sown in a sterilized peat moss–perlite substrate (3:1, v/v). Seedlings were grown under greenhouse conditions (25–28 °C, 25– 112 30% relative humidity) under continuous light and irrigated with one-tenth- strength Hoagland’s nutrient solution throughout the experimental period.

### 2.4 Plant growth-promoting rhizobacteria inoculum and conjugation workflow

*Stenotrophomonas rhizophila* strain GSB-381 (GenBank accession MK161197) was cultured in nutrient broth at 31 °C and 125 rpm until OD_600_ = 0.1 (≈10^8^ CFU mL^-^¹). For AuNP conjugation, equal volumes of bacterial suspension and phycosynthesized -AuNPs (1 mg mL^-^¹) were mixed and incubated at 31 °C for 30 min with gentle agitation (100 rpm), allowing natural association mediated by algal polysaccharide capping. The conjugation was confirmed as mentioned in section 2.2.

Sr and Sr-AuNP conjugates inoculation were performed at the dicotyledonary stage by applying 10 mL of bacterial suspension (10^8^ CFU mL^-^¹) to the root zone. Pathogen challenge was conducted two weeks later. Plants were maintained under a 16 h light/8 h dark photoperiod.

### 2.5 Phytopathogen target culture and preparation

*Erwinia persicina* USTRW7 (GenBank accession KU923347) was cultured on nutrient agar at 31 °C for 24 h. A single colony was transferred to nutrient broth and grown to OD_600_ = 0.5 (≈10^8^ CFU mL^-^¹) prior to plant inoculation.

### 2.6 Experimental design, treatments, and sample randomization

Experiments were conducted using a randomized complete block design with four treatments comprising 20 plants per treatment.

- T1: control (mock-inoculated with sterile distilled water).
- T2: *Stenotrophomonas rhizophila* + *E. persicina*.
- T3: AuNP-conjugated *S. rhizophila* + *E. persicina*.
- T4: *E. persicina* only.

#### 2.6.1 Biological replication and experimental reproducibility

Metabolomic analyses were performed on five biological replications for each treatment (n = 5). Each biological replicate consisted of pooling material from three individual plants to minimize intra-treatment variability and the amount of biomass required for the downstream multi-platform analyses.

#### 2.6.2 Randomization and quality control

A completely randomized design was employed in the greenhouse to eliminate positional effects. To prevent systematic bias during downstream processing, sample extraction and data acquisition (GC/MS, LC-MS/MS and ^1^H NMR) were performed in a randomized sequence. This approach ensured that instrumental drift or batch-specific variations were stochastically distributed across all treatments, maintaining the integrity of the comparative analysis.

#### 2.6.3 Robustness and statistical validation of the integrated dataset

To ensure the robustness of the integrated dataset comprising five samples (n=5) and to address the impact of high-dimensional noise, a comprehensive multi-tiered validation workflow was implemented.

Technical reproducibility: Metabolic features were screened based on a Relative Standard Deviation (RSD) threshold of < 20% across biological replicates. Only features demonstrating high technical stability were retained for downstream analyses.

### 2.7 Rhizosphere root exudate isolation and collection

Root exudates were collected under sterile conditions to minimize microbial growth during the incubation period. The use of sterile distilled water instead of CaCl_2_ followed the protocol of Fortier et al. (2023) with minor modification to avoid salt-derived spectral interference in downstream metabolomic analyses. Root systems remained intact and visually viable throughout the collection period. After incubation, exudates were immediately filtered (0.22 µm), lyophilized (Freeze Dryer, Labconco), and stored at −80 °C.

### 2.8 Untargeted metabolite profiling

#### 2.8.1 GC–MS analysis

Metabolite extracts (10 mg of freeze-dried root exudate) were derivatized by methoximation followed by silylation and analyzed using a Shimadzu GC–MS 2030 system [Analysis method: US-EPA 8270D (Rev.4, Feb.2007; U.S. Environmental Protection Agency, 2014)] equipped with a 5MS capillary column (30 m × 0.25 mm, 0.25 µm). The injector temperature was 250 °C, with helium as the carrier gas at 1 mL min^-^¹. The oven program was 70 °C (2 min), ramped 156 to 300 °C at 10 °C min^-^¹ and held for 5 min. Electron ionization mass spectra were acquired over m/z 50–600. Metabolites were annotated using the NIST library and reference standards were available.

#### 2.8.2 LC–MS/MS analysis

Liquid chromatography–tandem mass spectrometry was performed using a Waters 16 Xevo G2-S QToF system coupled to an Acquity UPLC with a BEH C18 column (2.1 × 163 100 mm, 1.7 µm) maintained at 40 °C. The mobile phase consisted of water (A) and 164 acetonitrile (B), both containing 0.1% formic acid. A linear gradient from 10% to 90% B was applied over 20 min. Mass spectra were acquired in negative electrospray ionization mode (200–1550 Da) using MSE acquisition. Leucine–enkephalin was used as the lock mass for mass accuracy correction.

#### 2.8.3 ¹H NMR spectroscopy

¹H NMR spectra were recorded on a Bruker 400 MHz spectrometer. Freeze-dried root exudate samples were dissolved in Deuterium oxide (D_2_O), and spectra were processed using standard Fourier transformation, phase correction, and baseline correction. Chemical shifts were referenced to solvent signals. Spectral analysis focused on relative signal intensities and chemical shift distributions corresponding to abundant low-molecular-weight metabolites.

#### 2.8.4 FTIR spectroscopy

Freeze-dried root exudate residues (5–10 mg) were homogenized and analyzed by FTIR spectroscopy using a Jasco FT/IR-6300 spectrometer equipped with an attenuated total reflectance (ATR) accessory. Spectra were collected over 4000–450 cm^-^¹ at 4 cm^-^¹ resolution with 32 co-added scans per sample. Background spectra were recorded prior to each run. Spectra were baseline-corrected and normalized prior to analysis. Functional group assignments were based on established IR band regions corresponding to carbohydrates, organic acids, amino acids, lipids, phenolics, and proteinaceous compounds. Comparative analysis focused on relative peak shifts and transmittance changes among treatments (Kainat et al., 2022).

### 2.9 Individual platform data processing and quality control

Integrated metabolomic analyses were performed using GC–MS, LC–MS/MS, ^1^H NMR, and FTIR datasets generated from tomato root exudate samples collected from four treatment groups: T1 (control), T2 (Sr+Ep), T3 (Sr-AuNP+Ep), and T4 (Ep). Each treatment consisted of five biological replicates, resulting in a total dataset of 20 samples.

#### 2.9.1 GC–MS chromatographic deconvolution

GC–MS chromatograms were subjected to baseline correction, peak detection, deconvolution, and retention time alignment prior to metabolite extraction. Peak abundances were normalized relative to total ion chromatogram intensity and expressed as relative percentage abundance. Detected metabolites included lipid methyl esters, Benzoic acid derivatives, sugars, and long-chain hydrocarbons.

#### 2.9.2 LC–MS/MS feature extraction

LC–MS/MS raw data were processed through feature detection, peak alignment, and deconvolution to generate an ion feature matrix based on mass-to-charge ratio (m/z) and retention time (RT). Features showing inconsistent retention time alignment or poor reproducibility across biological replicates were excluded prior to downstream analysis.

#### 2.9.3 ^1^H NMR spectral binning and integration

^1^H NMR spectra were manually phase- and baseline-corrected and subsequently segmented into chemically relevant spectral regions. Integrated spectral bins corresponding to lipid-associated aliphatic signals (δ 0.70–1.40 ppm), organic acid/amino acid-associated regions (δ 1.70–2.00 ppm), carbohydrate/oxygenated metabolite regions (δ 3.10–4.10 ppm), and aromatic regions (δ 8.30–8.50 ppm) were normalized prior to statistical analysis.

#### 2.9.4 FTIR spectroscopic processing and macromolecular surface clustering

**Figure 1:**
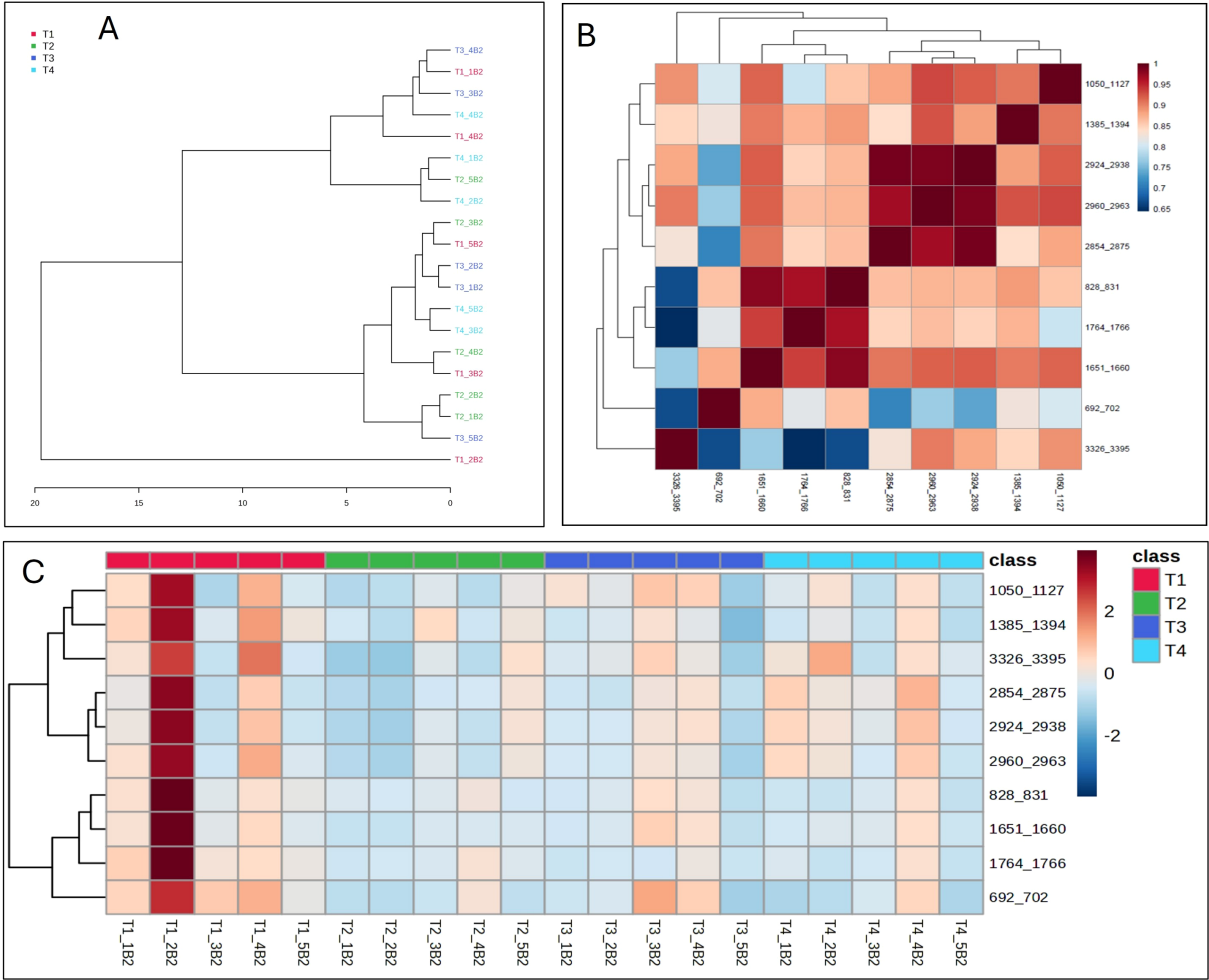
Multivariate statistical analysis of FTIR spectral profiles tracking chemical remodeling on the root surface and exudates. **(A)** Hierarchical clustering dendrogram illustrating the structural relationships and grouping patterns of tomato root surfaces across different treatments (T1, T2, T3, and T4). **(B)** Correlation matrix heatmap evaluating the degree of covariance and clustering behavior among key FTIR fingerprint regions, highlighting changes in lipids (e.g., asymmetric/symmetric CH_2_ stretching at 2924–2938 cm^-1^ and 2854–2875 cm^-1^, cell wall polysaccharides, and pectin esters. **(C)** Hierarchical clustering heatmap displaying the distinct intensity distribution profiles of selected spectral features across individual replicates of all experimental treatments. Absolute spectral data were processed and extracted using a Jasco FT/IR-6300 spectrometer (Jasco Inc., Easton, MD, USA).

For surface structural and macromolecular analysis, Fourier-Transform Infrared (FTIR) spectra of tomato root surfaces and corresponding exudates were recorded using a Jasco FT/IR-6300 spectrometer (Jasco Inc., Easton, MD, USA) equipped with an Attenuated Total Reflectance (ATR) accessory. Raw spectra were subjected to advanced ATR algorithmic correction to compensate for wavelength-dependent variations in depth of penetration. Then, followed by automatic baseline adjustment and min-max normalization to the most stable skeletal vibration peak. To assess chemical remodeling of the protective root cell wall barrier, the relative intensities of aliphatic lipid bands (2850 and 2920 cm^-1^) and ester-linked carbonyl networks (1730–1745 cm^-1^) were quantified. From these processed profiles, 10 prominent, characteristic spectral features representing key structural components (including lipid CH_2_ stretches, cell-wall structural polysaccharides, and pectin esters) were extracted from the 20 samples (n = 5 biological replicates per treatment group). This dedicated spectral dataset was utilized exclusively for the independent evaluation of root surface structural modifications. The extracted feature matrix was analyzed to evaluate macromolecular divergence. This analysis successfully identified distinct, treatment-specific clustering patterns. It represents the fingerprint region (1800–600 cm^-1^). The analysis used independent unsupervised hierarchical clustering analysis (HCA) and correlation evaluation using MetaboAnalyst 6.0. Clustering was executed utilizing a Euclidean distance metric and a complete linkage sorting algorithm. This generated the final structural dendrogram, correlation matrix, and intensity profiles presented in **Figure 1**.

### 2.10 Integrated multi-platform chemometrics and pathway topology

Following individual platform processing as described in sections 2.9.1 to 2.9.3, datasets from all analytical platforms were integrated into a unified quantitative feature matrix consisting of samples as rows and metabolite features as columns. As detailed in the previous sub-sections, this high-dimensional dataset was constructed using stringent filtering criteria. This included the systematic removal of low-abundance and low-variance variables as well as the exclusion of poorly reproducible features. Signals consistently detected across all biological replicates and treatments were retained. This filtering step ensured a robust, noise-free data matrix for downstream multivariate analysis.

The final integrated dataset consisted of 20 samples × 27 metabolomic features, including GC–MS-derived metabolites, LC–MS/MS ion features, and selected ^1^H NMR spectral regions. Integrated statistical analyses were conducted using the Statistical Analysis (One-Factor) module in MetaboAnalyst 6.0 following normalization by sum and autoscaling to reduce systematic intensity bias while preserving relative biological variation.

#### 2.10.1 Cross-platform data integration and preprocessing

Unsupervised principal component analysis (PCA) was initially performed to evaluate overall clustering patterns and identify potential outliers among treatments. Statistical identification of treatment-associated metabolite features was subsequently performed using one-way ANOVA followed by false discovery rate (FDR) correction and Tukey’s honestly significant difference (HSD) *post hoc* analysis. Metabolite features with FDR-adjusted significance values (q < 0.05) were considered statistically associated with treatment-dependent variation and were retained for downstream pathway and integrative analyses.

#### 2.10.2 Multivariate and univariate statistical analysis

To visually assess global cross-platform metabolic profiles and treatment group relationships, hierarchical clustering analysis (HCA) was executed using an agglomerative hierarchical algorithm based on Euclidean distance and Ward’s linkage method. A bidirectional heatmap was generated using a standard Z-score transformation, calculated as:


Z=(x−µ)/σ

where x represents the individual feature intensity, µ is the mean abundance of the feature across all samples, and σ is the corresponding standard deviation, thereby standardizing multi-platform abundances across a uniform scale for downstream visualization.

#### 2.10.3 Biochemical network connectivity and secondary metabolism mapping

To evaluate the functional relationships and metabolic crosstalk between the integrated analytical platforms, biological features were mapped against established biochemical networks. Extracted metabolomic markers, prominent ^1^H NMR spectral regions, and functional macromolecular groups were contextualized using the Kyoto Encyclopedia of Genes and Genomes (KEGG; http://www.kegg.jp) and the Pathway Analysis module of MetaboAnalyst 6.0. This mapping procedure was utilized to reconstruct interconnected metabolic networks. It enables visualization of secondary metabolic pathways, metabolic hubs, and systemic biochemical connectivity shifts triggered by the experimental treatments.

#### 2.10.4 Pathway enrichment and central carbon topology analysis

Treatment-associated features identified as statistically significant via univariate analysis (q < 0.05; see Section 2.10.2) were mapped onto the *Solanum lycopersicum* (tomato) pathway library using the MetaboAnalyst 6.0 Pathway analysis module. Pathway topology was calculated using relative-betweenness centrality to assess the “impact” of specific metabolites within the biological network. Functional mechanistic insights were derived by integrating the identified Very-Long-Chain Fatty Acids (VLCFAs) and their derivatives into the Cutin, Suberin, and Wax biosynthesis pathway (KEGG map00073; Kanehisa et al., 2006). This approach allowed for the reconstruction of the biosynthetic routes and enzymatic sequences involved in the conversion of long-chain acyl-CoA precursors into structural aliphatic monomers. The analysis focused on identifying pathway enrichment and the functional positioning of metabolites associated with the reinforcement of root physical barriers, providing a statistically grounded basis for the observed metabolic reprogramming.

Network-based pathway visualization was additionally performed using the Network Explorer module in MetaboAnalyst to examine relationships among lipid-associated and primary metabolism-related metabolite groups detected in the dataset.

#### 2.10.5 Metabolite annotation confidence and metabolomics standards initiative compliance

Putative metabolite identity assignment was performed in strict accordance with the reporting standards established by the Metabolomics Standards Initiative (MSI). Cross-platform chemical features were categorized into definitive confidence tiers based on the depth of orthogonal analytical data available. High-resolution LC-MS/MS features and GC-MS structural profiles were classified as MSI Level 2 (putatively identified compounds). These annotations were derived via automated spectral similarity matching against authenticated reference libraries (NIST). A minimum forward/reverse match threshold of 80% was used. The matches were based on diagnostic MS/MS fragmentation patterns and verifying retention index consistency. For primary metabolites evaluated via ^1^H NMR spectroscopy, structural assignments were designated as MSI Level 2. The assignments were based on chemical shift coupling profiles (δ, ppm). The profiles were matched against the Chenomx NMR Suite database (800 MHz library) and the Human Metabolome Database (HMDB). For complex structural assemblies or isolated infrared absorption bands, specific structural isomerism could not be definitively resolved. In such cases, annotations were restricted to MSI Level 3 (putatively characterized compound classes). In the absence of direct co-elution confirmation with commercial authentic reference standards for every individual detected feature, this multi-tier assignment framework is used. It ensures a highly transparent, standardized, and reproducible reporting index for the tomato rhizosphere dataset.

## 3 Results

### 3.1 Optical and structural verification of AUNP formation

**Figure 2:**
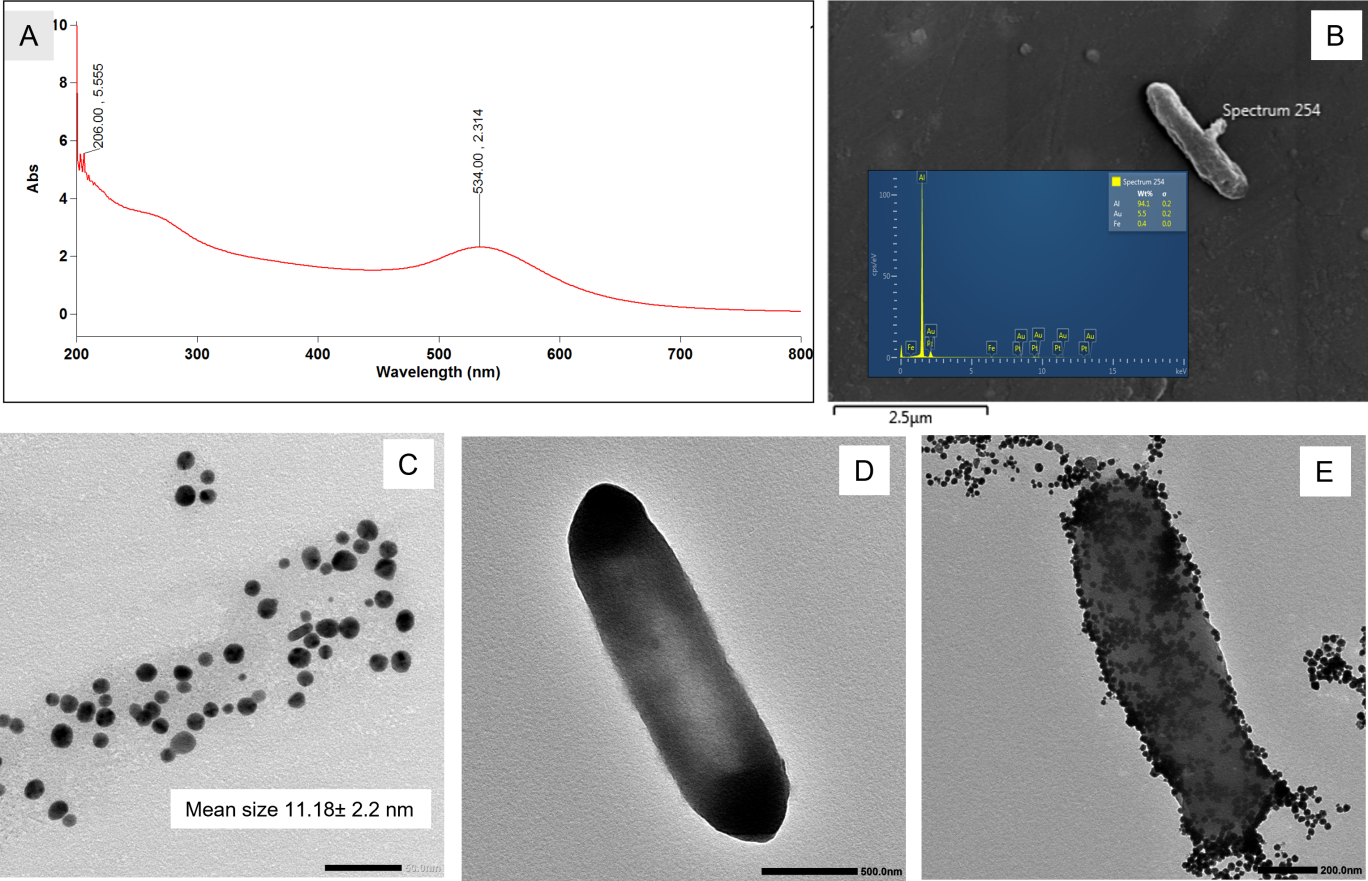
Characterization and PGPR-conjugation of phycosynthesized gold nanoparticles. **(A)** UV-Visible absorption spectrum of phycosynthesized AuNPs with an SPR peak at 534 nm. **(B)** SEM image and EDX spectrum showing gold deposition on the PGPR surface. **(C)** TEM micrograph of phycosynthesized AuNPs showing a mean size of 11.18 ± 2.2 nm. **(D)** TEM of native *Stenotrophonas rhizophila* (PGPR) cell (Control). **(E)** TEM micrograph of the phycosynthesized AuNP-PGPR conjugate, illustrating the dense clustering of nanoparticles on the bacterial cell wall, facilitating enhanced root adhesion. Scale bar: **(C)** 50 nm; **(D)** 500 nm, and **(E)** 200 nm. Magnification: X120k.

The UV–Visible spectrum indicated the presence of a surface plasmon resonance (SPR) peak at approximately 534 nm, representing successful formation of stable AuNPs (**Figure 2A**). The increase in absorbance intensity with increasing precursor concentration corresponds to the nanoparticle yield and shows better control of the synthetic synthesis conditions during fabrication.

### 3.2 Morphological evidence for conjugation

**Figure 3:**
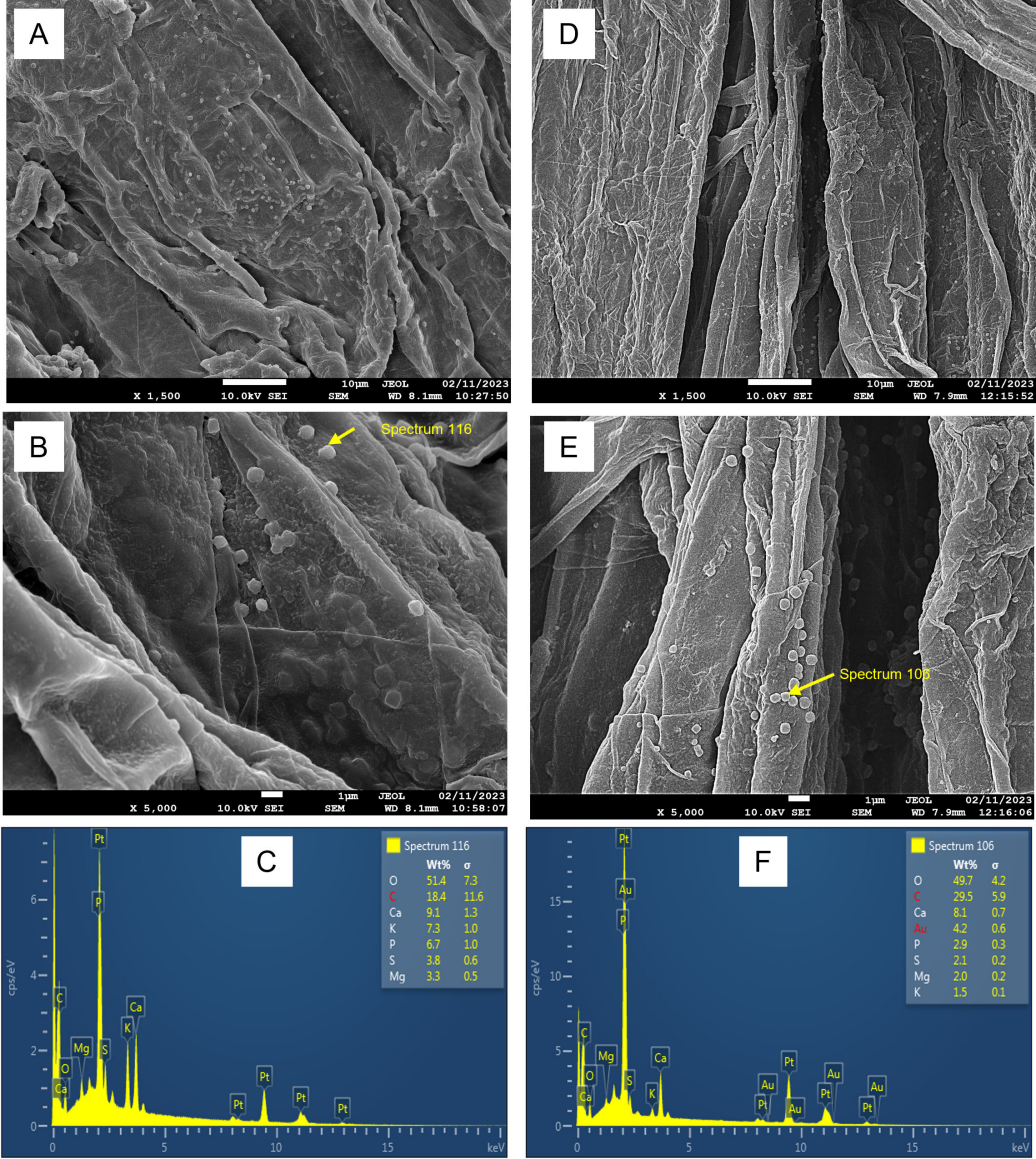
Comparative SEM-EDX analysis of tomato root colonization by *Sr* and *Sr*-AuNP conjugates. Scanning Electron Microscopy (SEM) and Energy Dispersive X-ray (EDX) micrographs illustrating the differential colonization and elemental profiles on tomato root surfaces. **(A–C)** T2 group (*Sr* non-conjugates): **(A)** Representative root surface morphology at 1,500X magnification (Scale bar: 10 µm). **(B)** High-magnification micrograph at 5,000X (Scale bar: 1 µm) showing standard rhizoplane colonization by non-conjugated *Stenotrophomonas rhizophila*. **(C)** Corresponding EDX spectrum (Point 116) confirming a native elemental profile (C, O, Ca, K) with a complete absence of gold. **(D–F)** T3 group (*Sr*-AuNP conjugates): **(D)** Root architecture at 1,500X (Scale bar: 10 µm) following application of the bio-nanocomposite. **(E)** High-resolution image at 5,000X (Scale bar: 1 µm) revealing dense, spherical nanoparticle clusters anchored to the bacterial-root interface. **(F)** EDX spectrum (Point 106) providing definitive elemental confirmation of Gold (Au; 4.2 mass%) localized at the colonization site, distinct from the Al and Pt background peaks. All images were captured at an accelerating voltage of 10.0 kV.

Transmission electron microscopy (TEM) photographs (**Figure 2C–E**) showed that predominantly spherical AuNPs with a mean diameter of 11.18 ± 2.2 nm (**Figure 2C**) were present on bacterial surfaces (**Figure 2E**). By comparison, *S. rhizophila* cells which were not treated were smooth and had a rod shape (**Figure 2D**), whereas the cells with AuNPs conjugated to their surfaces had increased density of AuNPs on the cell surface (**Figure 2E**). This indicates that there was successful conjugation of AuNPs to bacterial cells. The results of field-emission scanning electron microscopy – energy dispersive X-ray spectroscopy (FESEM–EDS) shown in **Figures 2B**, **3** were also consistent with this result; a strong signal for gold (~5.5 wt%; 4.2 wt%, respectively) (**Figure 2B** inset) was clearly identified in all conjugated samples, indicating AuNPs were in close proximity to bacterial cells.

### 3.3 Dynamic light scattering and zeta potential analyses

The dynamic light scattering (DLS) studies showed that the hydrodynamic diameter of the conjugated AuNPs was significantly larger than that of the AuNPs themselves (from 78.15 ± 10.89 nm to 90.96 ± 1.96 nm) compared to that of untreated bacteria alone (1028.33 ± 39.55 nm). This suggests that the AuNPs were adsorbed onto the surface of the bacterial cells. The polydispersity index (PDI) values (0.26–0.34) confirmed that there was moderate stability of dispersion in all samples. Zeta potential analysis showed that the zeta potentials of the AuNPs (−21.37 ± 1.52 mV) and untreated bacteria (−39.55 ± 1.80 mV) shifted closer to each other (−28.56 ± 1.94 mV) for the conjugates, indicating that both electrostatic interactions and overall stability were a result of the formation of the hybrid material (**Table 1**).

**Table 1:** Comparative physicochemical and elemental characterization of phycosynthesized AuNPs, *Stenotrophomonas rhizophila* (*Sr*), and their bio-nanocomposite conjugates.

Techniques	AuNPs	T2: Sr (non-conjugates)	T3: Sr-AuNPs (conjugates)
Z-average (nm)	78.15 ± 10.89	1028.33 ± 39.55	90.96 ± 1.96
Poly disperse intensity (Pdi)	0.26 ± 0.01	0.34 ± 0.12	0.33 ± 0.05
Zeta potential (mV)	-21.37 ± 1.52	-39.55 ± 1.80	-28.56 ± 1.94
Major FTIR peaks (cm^−1^)	3248, 1627, 1416, 1025	3273, 1626, 1532, 1233	3279, 1630, 1536, 1037
Peaks interpretation	Polyphenolic OH, C=C stretch	Amide I & II, Carbohydrate C-O	Surface functionalization shift
ICP-MS Au (ppb)	45779.08 ± 1205.31	0.45 ± 0.05	3891.88 ± 115.05

### 3.4 FTIR analysis of the surface functionalization of AuNPs

The FTIR spectra recorded for AuNPs exhibited four noticeable peaks distinguishing -OH and C=C stretching vibrations in Polyphenolic compounds. The characteristic bands on *S. rhizophila* are also present in FTIR spectra, which will correlate with Amide types I and II, Protein amount recorded as 3273, 1626, 1532, & 1233 cm^-^¹ as well as a C-O structural component found in carbohydrates.

There was a noticeable shift in the peak positions (to 3279, 1630, 1536, and 1037 cm-¹ respectively) of the FTIR spectra obtained for Sr-AuNP conjugates, suggesting that both proteinaceous and polysaccharide functional groups were involved in binding the nanoparticles to the bacterial cells (**Table 1**).

### 3.5 Quantification of the Au content in the Sr-AuNP conjugates

The ICP-Mass Spectrometry results indicated that the amount of Au within the Sr-AuNP Conjugates was 3891.88 ppb as opposed to the negligible Au present within the bacterial controls (0.45 ppb); this confirms that the AuNPs were successfully loaded onto the bacterial cells (**Table 1**).

### 3.6 Conceptual framework of root-pathogen-nano interactions

**Figure 4:**
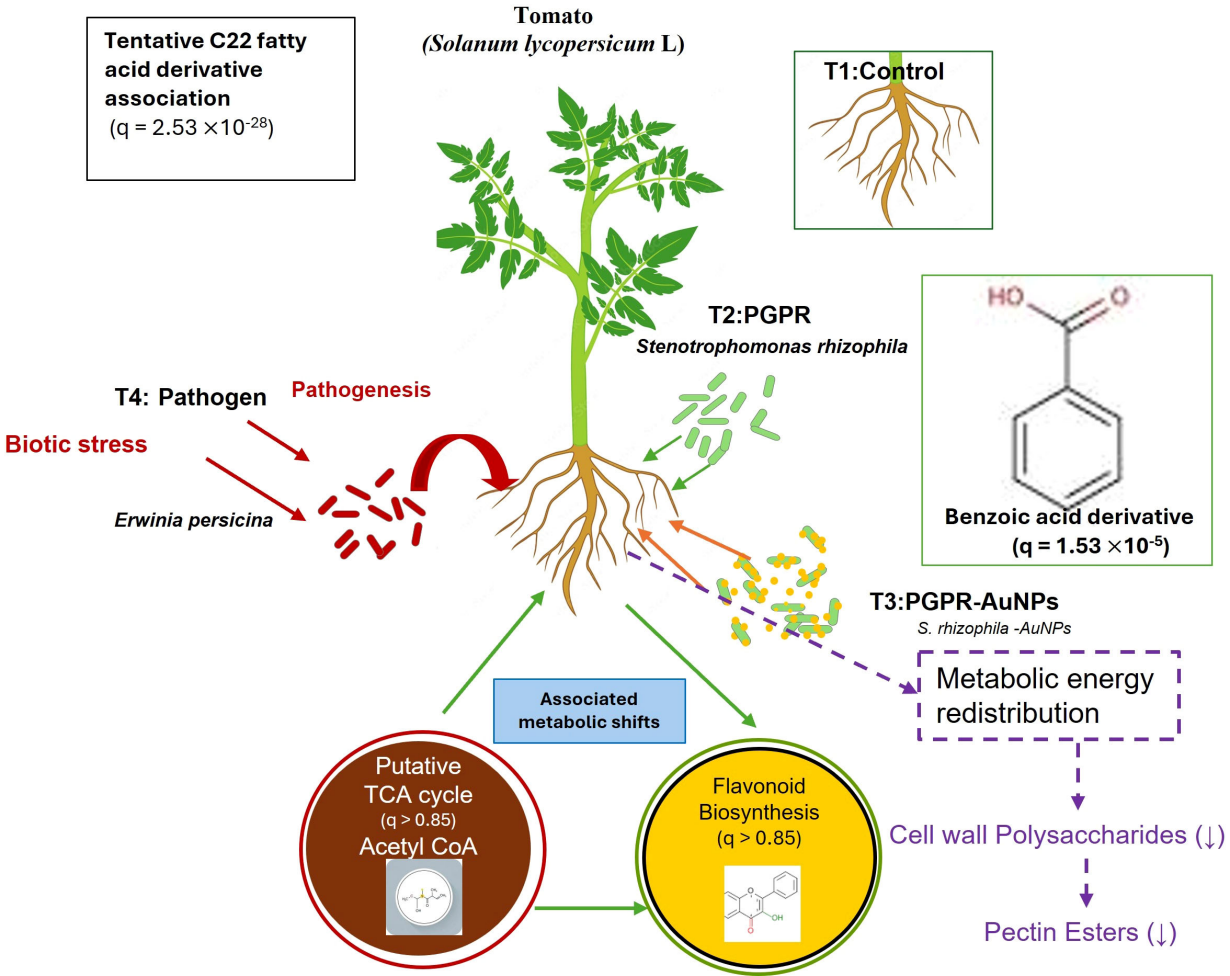
Conceptual framework summarizing treatment-associated metabolite patterns in tomato root exudates. Schematic overview of metabolite features associated with tomato (*Solanum lycopersicum*) roots following challenge with *Erwinia persicina* (T4: Ep) and treatments with *Stenotrophomonas rhizophila* (T2: Sr+Ep) or Sr–AuNP conjugates (T3: Sr-AuNPs+Ep). Putatively identified lipid amides and Benzoic acid derivatives are highlighted as metabolites associated with treatment-specific metabolite profiles, with statistical significance reported as FDR-adjusted p-values (q). Dashed arrows indicate hypothesized associations among metabolite groups identified through pathway topology analysis. Pathways including the Citrate (TCA) cycle and Flavonoid biosynthesis represent inferred functional associations derived from MetaboAnalyst 6.0 analysis and do not indicate direct measurements of pathway activity or metabolic flux. Chemical structures were obtained from the NIST Chemistry WebBook; schematic prepared using Microsoft PowerPoint.

The integration of GC–MS, LC–MS/MS, and ^1^H NMR datasets revealed distinct, treatment-dependent metabolic trajectories (**Figure 4**). The rhizosphere of plants challenged by *Erwinia persicina* (T4) was characterized by a robust stress-dominant profile. In contrast, the bionanocomposite treatment (*S. rhizophila*-AuNP, T3) induced a specialized metabolic reconfiguration distinct from both the control (T1) and native rhizobacterial priming (T2).

Univariate statistical inference using one-way ANOVA with False Discovery Rate (FDR) correction (q < 0.05) was deployed to identify the specific discriminant metabolites driving the observed treatment-specific clustering separation (**Figure 4**; **Table 2**).

**Table 2:** Identification of metabolic biomarkers in tomato root exudates across multiple platforms.

Platform	Feature	Putative identification	MSI level	Associated pathway	FDR (q-value)	Tukey’s HSD (*p <* 0.05)
LC-MS/MS	m/z 338.33	Tentative lipid amide (C22)	2	Cutin, suberin and wax biosynthesis	2.53 × 10^-28^	T3-T1; T4-T1; T3-T2; T4-T2; T4-T3
LC-MS/MS	m/z 337.06	Long-chain lipid ion	3	Cutin, suberin and wax biosynthesis	1.89× 10^-13^	T2-T1; T4-T1; T3-T2; T4-T2; T4-T3
GC-MS	m/z 270.31	Oleanitrile	2	Biosynthesis of unsaturated fatty acids	4.19× 10^-4^	T2-T1; T3-T2; T4-T2
GC-MS	GC_BA-TMS	Benzoic acid derivative	2	Phenylpropanoid/ Flavonoid biosynthesis	1.53× 10^-5^	T3-T1; T4-T1; T3-T2; T4-T2
^1^H NMR	δ 0.70–1.40 ppm	Lipid-associated regions	3	Specialized Fatty acid metabolism	7.75× 10^-3^	T3-T1; T3-T2; T4-T3
^1^H NMR	δ 2.4–2.6 ppm	Citrate/Succinate	2	Citrate cycle (TCA cycle)	< 0.05	T2-T1; T3-T2
^1^H NMR	δ 6.5–7.5 ppm	Phenolic derivatives	3	Flavonoid biosynthesis	< 0.05	T2-T1; T3-T2

Metabolite Identification (MSI) levels: 2, putatively identified compounds (based on spectral matches with public/commercial libraries); 3, putatively identified compound classes. Statistical significance is reported as FDR-adjusted p-values (q) derived from one-way ANOVA. Tukey’s HSD denotes significant pairwise differences between treatment groups: Control (T1), *S. rhizophila* (T2), *S. rhizophila*-AuNP (T3), and *E. persicina* (T4).

The primary multi-platform biomarker driving treatment-specific variance was a tentative lipid amide (m/z 338.33), which exhibited an extreme differential abundance across groups (q = 2.53 ×10^-28^). Tukey’s *post hoc* HSD analysis confirmed that this lipid amide marker was heavily accumulated in both the *Erwinia persicina*-challenged host roots (T4) and the protective, nanoparticle-primed Sr-AuNPs roots (T3) relative to the untreated controls. Consistent this accumulation profile, a highly correlated secondary mass feature, m/z\ 337.06, putatively characterized as a very-long-chain fatty acid (VLCFA) derivative, also demonstrated high statistical significance (q = 1.89 × 10^-13^). The concurrent upregulation of these two distinct aliphatic markers firmly establishes hydrophobic, long-chain lipid signaling as a primary metabolic response coordinated at the host rhizosphere interface during both pathogen challenge and nano-bio priming interactions.

Pathway topology analysis identified the Citrate (TCA) cycle and Flavonoid biosynthesis as high-impact metabolic nodes within the dataset. While these represent preliminary functional associations (q > 0.85 for pathway impact), they suggest a systemic redistribution of resources. This is corroborated by the observed reduction in cell wall polysaccharide and pectin ester signals in T3 and T4, supporting a “metabolic trade-off” hypothesis where energy is diverted from primary growth to defense-associated biosynthesis.

FTIR analysis supported these findings, showing treatment-dependent shifts in carbohydrate- and lipid-associated spectral regions, consistent with the coordinated metabolic reconfiguration observed via MS and NMR platforms.

### 3.6 Integrated multivariate exploration and biomarker-associated features

**Figure 5:**
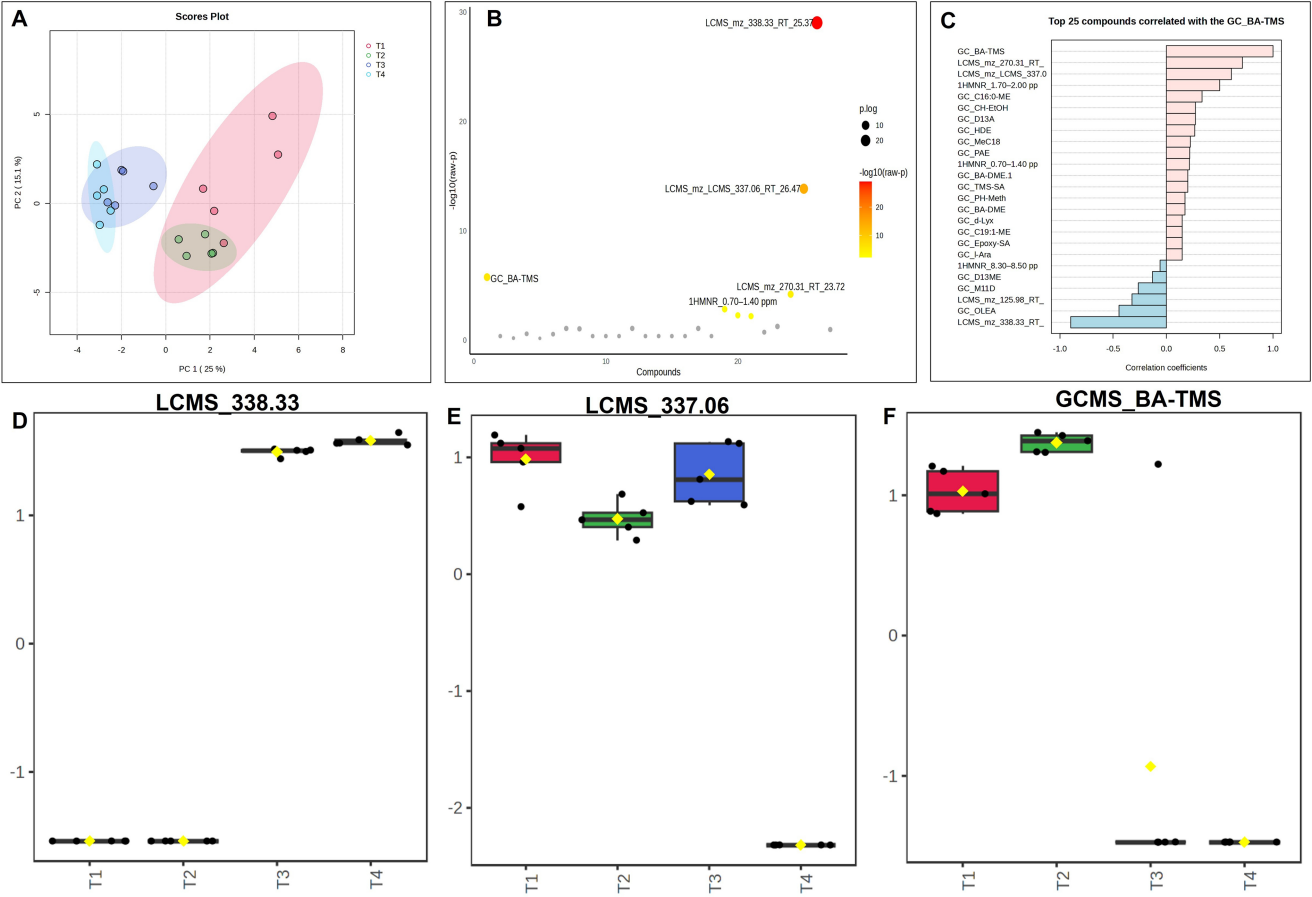
Integrated multivariate exploration and univariate statistical inference of the tomato rhizosphere metabolome. **(A)** Unsupervised principal component analysis (PCA) score plot revealing distinct, treatment-dependent metabolic reprogramming along PC1 (25%) and PC2 (15.1%). Replicates for treatments T3 (dark blue) and T4 (light blue) cluster tightly together in the negative PC1 space away from control group T1 (pink color), showing a pronounced shift in the total rhizosphere metabolic profile (n = 5 biological replicates per group; shaded areas denote the 95% confidence ellipses). **(B)** Univariate feature significance plot mapping the -log10 across all 27 integrated analytical features, identifying LCMS_338.33, LCMS_337.06, and GC_BA-TMS as the primary drivers of experimental variance exhibiting the highest statistical significance. **(C)** Feature correlation profile (Pattern Hunter) identifying the top 25 features strongly associated with the principal marker GC_BA-TMS. Features in pink show positive covariance, while features in blue display an inverse relationship, indicating coordinated shifts between distinct metabolic pathway branches. **(D–F)** Relative abundance distribution box plots of the top three significant markers highlighting divergent accumulation patterns: LCMS_338.33 acts as a highly specific biomarker exclusively upregulated in treatments T3 and T4, while LCMS_337.06 and GC_BA-TMS exhibit suppressed or fluctuating dynamics in response to specific treatment applications. Black dots represent individual biological replicates, and yellow diamonds indicate group means. Statistical significance was determined using one-way ANOVA followed by Tukey’s honestly significant difference (*post hoc* HSD) test under a False Discovery Rate threshold (q < 0.05).

To assess the global metabolic shifts in the tomato rhizosphere across treatment groups (T1–T4), an unsupervised Principal Component Analysis (PCA) was performed. The PCA score plot revealed distinct clustering patterns, indicating that the presence of *S. rhizophila*, particularly when conjugated with AuNPs (T3), induced a natural metabolic reprogramming. This reprogramming was distinct from the control (T1) and pathogen-challenged plants (T4) (**Figure 5A**). Importantly, this separation was observed without supervised bias, ensuring the clusters represent inherent biological variance.

To identify the biochemical factors driving this differentiation with high statistical rigor, a one-way ANOVA with FDR correction was applied to the integrated data from LC-MS/MS, GC-MS, and ^1^H NMR platforms. The resulting Manhattan plot (**Figure 5B**) identified several highly significant metabolic hubs:

#### 3.6.1 Stress-induced lipid amide accumulation

The tentative lipid amide (m/z 338.33) was the most significant feature (*p <* 10^-28^), showing prominent accumulation in T3 and T4 samples as a marker for biotic stress.

#### 3.6.2 Phenolic partitioning and correlation with lipid derivatives

Quantitative analysis showed that Benzoic acid (GC_BA-TMS) levels were significantly higher in T2 compared to all other treatments. Conversely, inter-platform correlation analysis demonstrated a strong mirrored negative or inverse correlation (≈-0.9) between Benzoic acid and the C22 fatty acid derivative (**Figure 5C**). In roots with Sr-AuNP (T3), Benzoic acid levels were notably lower, while Very-Long-Chain Fatty Acid (VLCFA) derivatives were significantly elevated (q < 0.05).

#### 3.6.3 Resource reallocation and metabolic signaling

Significant reductions in basal structural components herein referred to as homeostatic markers were noted across treated groups. Specifically, Pentanedioic acid, di(2,4-di-t-butylphenyl) ester (PAE) and a Hexadecanoic acid, 1-(hydroxymethyl)-1,2-ethanediyl ester (GC_HDE), which normally regulate cell membrane maintenance under healthy conditions, were markedly suppressed in both the Sr-AuNPs-primed (T3) and *Erwinia persicina*-challenged (T4) roots compared to healthy T1 controls. This drop supports a “metabolic trade-off” hypothesis, where the host shifts restricted carbon resources away from routine maintenance toward the high energy demands of defensive signaling. Interestingly, the triterpenoid defense precursor Oleanitrile (m/z 270.31) was stabilized in T3 compared to the depleted T4 group, indicating a specialized “nano-bio” metabolic phenotype that mitigates total homeostatic collapse.

The mathematical robustness of these profiles was cross-validated using a non-parametric Kruskal-wallis test followed by Dunn’s *post hoc* analysis. This parallel validation confirmed that the abundance of both the upregulated defense biomarkers and the downregulated structural homeostatic markers was significantly altered (q < 0.05) in response to the multi-kingdom interactions between tomato roots, *S. rhizophila*, and *E. persicina*. This provides a rigorous, distribution-independent basis for the observed metabolic differentiation (**Figure 5D**).

### 3.7 Hierarchical clustering and population structure

To evaluate systemic metabolic relationships across treatments, unsupervised hierarchical clustering analysis (HCA) was performed on the integrated cross-platform dataset (comprising GC-MS, LC-MS/MS, and ^1^H NMR profiles, excluding surface FTIR features).

**Figure 6:**
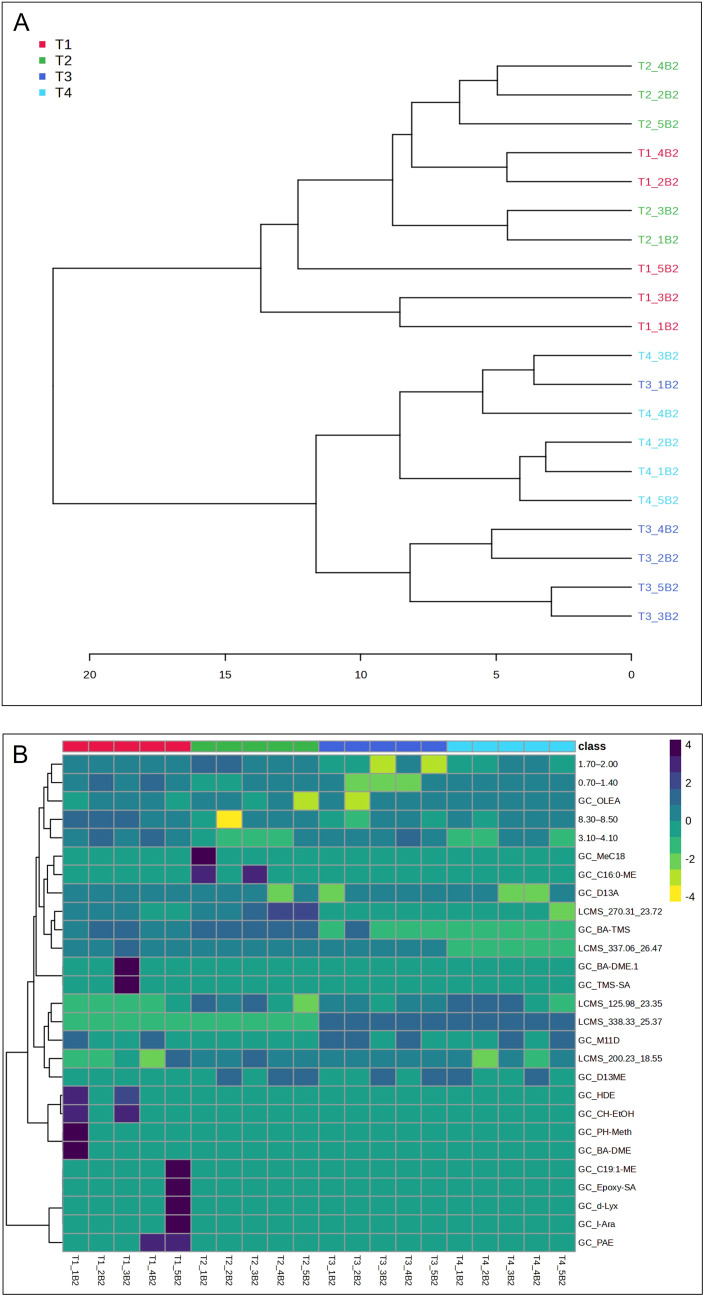
Hierarchical clustering analysis (HCA) and heatmap of differential metabolic profiles. **(A)** Dendrogram of HCA: Global similarity analysis between treatment groups (T1 to T4). The clustering was performed using the Euclidean distance method with the horizontal axis representing the levels of dissimilarity. The primary branching demonstrates a clear metabolic convergence between the bionanocomposite (T3) and pathogen-challenged (T4) groups. **(B)** Heatmap of differential metabolic features: Visualization of relative abundance for the top discriminant markers. Data were normalized using Z-score transformation, where dark purple indicates high relative abundance and yellow indicates low relative abundance. Organization: Rows represent integrated chemical features and spectral windows from LC-MS, GC-MS, and ^1^H NMR platforms. Columns stand for individual biological replicates (n = 5 per group). Color Coding: T1 (Control) - red; T2 (*S. rhizophila*) - green; T3 (Sr-AuNP) - dark blue; T4 (*E. persicina*) - light blue.

The HCA generated a dendrogram (**Figure 6A**), which shows that the tomato rhizosphere metabolome clusters into two distinct main clades. The first clade consisted largely of the untreated control (T1) and *S. rhizophila*-primed (T2) groups and indicates that they have an established (baseline) metabolic architecture that exists independently of the pathogen activity observed in their respective metabolomes.

The clustered heatmap in **Figure 6B** identifies discrete functional blocks that differentiate the treatments as described in the following subsections:

Suppression of baseline metabolites: A significant reduction in homeostatic markers, including GC_PAE, 8.30–8.50 ppm, and 3.10–4.10 ppm, was observed in both T3 and T4 compared to the T1 control. This commonality likely represents a core metabolic trade-off as the host shifts resources from primary metabolism to defense-associated engagement.

Nanocomposite-specific induction: In contrast to the pathogenic response, the T3 group exhibited a distinct up-regulation in the 1.70–2.00 ppm region and specific Benzoic acid derivatives (e.g., GC_BA-DME). These markers were notably reduced or absent in the T4 group, identifying them as candidate signatures for the unique mechanism of action of the bio-nanocomposite.

Pathogen-responsive accumulation: Markers such as LCMS_338.33 (tentative lipid amide) and LCMS_337.06 showed the highest abundance in the T4 and T3 cohorts (**Figures 5E, F**). This validates their role as statistically robust indicators of the stress-induced metabolic state identified in our univariate analysis.

Stability of the primed state: The T2 group maintained high similarity to the T1 control across several clusters (e.g., cis-10-Nonadecenoic acid, methyl ester -GC_C19:1-ME, 5.alpha.,6.alpha.-Epoxy-17-oxo-6.beta.-pentyl-4-nor-3,5-secoandrostan-3-oicacid, methyl ester- GC_Epoxy-SA), indicating that native rhizobacterial priming induces a more subtle adjustment of the metabolic baseline compared to the radical reconfiguration triggered by AuNP-conjugation or pathogen infection.

### 3.8 FTIR-based clustering reveals treatment-specific chemical reconfiguration of tomato root surfaces and exudates

Hierarchical clustering dendrogram of individual biological replicates based on normalized FTIR peak intensity revealed well-defined structuring of the tomato root metabolome in response to different treatments (**Figure 1A**).

The correlation analysis of features indicated that the lipid and protein classes had co-ordination of overall positive association with one another, and conversely an overall negative association with the carbohydrate class. The metabolic reallocation patterns observed show the same pattern of treatment induced metabolic trade-offs that may be related to a defense mechanism (**Figure 1B**).

Heatmap-based visualization highlighted significant changes across different spectral regions. These patterns were clear and unique for each treatment. Aromatic- associated regions between 692 and 702 cm^-^¹ were found to be more dominant in T1. Carbohydrate-associated regions between 870 and 874 cm^-^¹, as well as 1050 and 1127 cm^-^¹, were found to be higher in both T1 and T3 but were lower in T2. Protein-associated regions between 1385 and 1394 cm^-^¹ and between 1651 and 1660 cm^-^¹ were found to be higher in T1 and T4 (**Figure 1C**). However, regions associated with lipid metabolism showed different patterns. The ester carbonyl and CH stretching regions between 1764 and 1766 cm^-^¹ were higher inT3 and T4. Similarly, regions between 2854 and 2875 cm^-^¹, and between 2924 and 2963 cm^-^¹, were also elevated in T3 and T4 but were lower in T2.

### 3.9 Biological annotation and confidence assessment of discriminant metabolites

**Figure 7:**
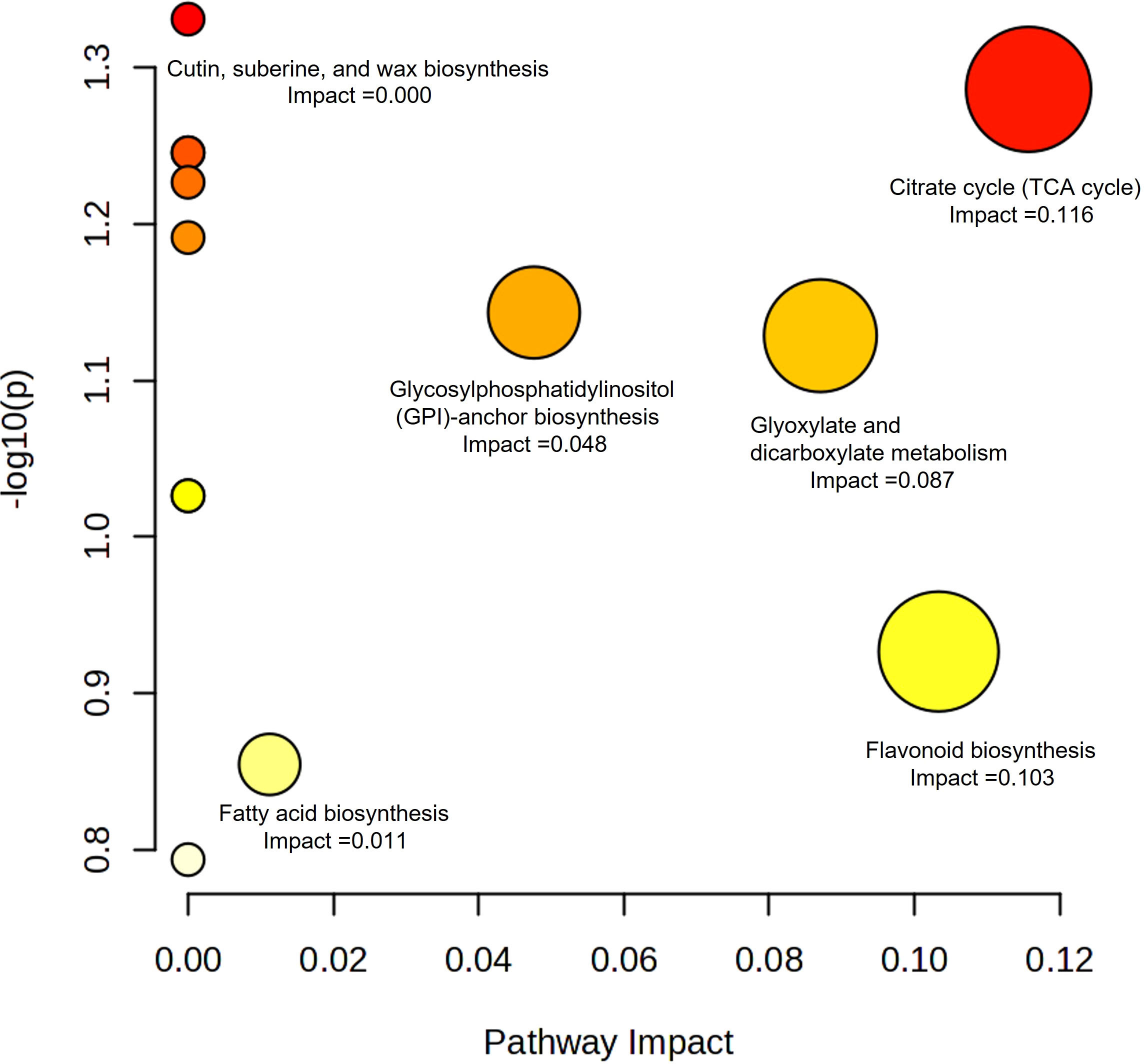
Multi-platform analysis of tomato root metabolic shifts under *Erwinia persicina* stress and bio-nanocomposite treatment. Metabolic pathway topology: Bubble plot illustrating the functional impact of differentially regulated metabolites. The y-axis shows pathway enrichment significance (-log_10_ *p* >), and the x-axis shows pathway impact based on network centrality. The Citrate cycle (TCA cycle) and Flavonoid biosynthesis represent the pathways with the highest topology impact among the detected metabolites (Impact > 0.1). Circle size denotes the impact score; color intensity (yellow to red) denotes statistical significance.

To provide functional context to the treatment-specific signatures identified in **Figure 7**, key discriminant features were annotated using the KEGG and HMDB databases. As detailed in **Supplementary Table 1** (derived from the Pathway name map), several identified metabolites are central to plant-microbe signaling and primary metabolism. Specifically, the rhizobacteria-associated marker GC_BA-TMS was mapped to Benzoic acid (C00180), a known intermediate in the phenylpropanoid pathway. Furthermore, the suppression of primary metabolic signals in the T3 and T4 groups corresponded to shifts in Citric acid (C00158) and L-Valine (C00183), while defense-related signaling was marked by the presence of Flavonoids such as Rutin (C05625) and Naringenin (C00509).

### 3.10 Multi-platform pathway topology and functional significance analysis

To evaluate the functional relevance of the discriminative metabolites, a combined pathway topology and enrichment analysis was performed using the *Solanum lycopersicum* library (KEGG organism code: sly) within the MetaboAnalyst 6.0 platform (**Table 3**). The pathway topology analysis provided further insight into the functional context in which these metabolite-level changes occur (**Figure 7**). Among these pathways, the Citrate (TCA) cycle had the highest pathway impact (0.115), which was closely followed by Flavonoid biosynthesis (0.103)., indicating these pathways to be key metabolically active pathways that respond to treatment-specific perturbation. According to the metabolic pathway enrichment, Cutin, Suberin and Wax biosynthesis had a significant alteration (P-value 0.046). This change was mainly influenced by the presence of lipid related signals providing strong evidence for the structural reinforcement role of 13-docosenamide in Sr-AuNP-primed plants. The topology effect may not be high but from the statistical analysis, the pathway is indeed important in terms of functionality. Notably, the Cutin, Suberin, and Wax biosynthesis pathway was the most statistically significant (*p <* 0.05). Glyoxylate and dicarboxylate metabolism (0.087) and GPI anchor biosynthesis (0.048) had intermediate pathway impacts, while fatty acid biosynthesis had a lower pathway impact (0.011) (**Table 3**). Overall, these results suggest that both pathogen stress and nano-elicitor treatment specifically modulate central carbon and secondary metabolite biosynthesis pathways rather than lipid biosynthesis pathways.

**Table 3:** Pathway topology and enrichment analysis of tomato (*Solanum lycopersicum*) root exudate metabolites conducted via MetaboAnalyst 6.0 using the *Solanum lycopersicum* (sly) library.

Metabolic pathway	KEGG ID	Hits	Raw p	Impact	Functional role in defense
Cutin, suberin, and wax biosynthesis	sly00073	1	0.0466	0.000	Structural barrier and physical reinforcement
Citrate cycle (TCA cycle)	sly00020	1	0.0517	0.115	Energetic pivot for systemic resistance
Flavonoid biosynthesis	sly00941	1	0.1184	0.103	Synthesis of antimicrobial secondary metabolites
Glyoxylate and dicarboxylate metabolism	sly00630	1	0.0743	0.087	Stress adaptation and carbon recycling
Glycerolipid metabolism	sly00561	1	0.0611	0.117	Membrane remodeling and lipid signaling
GPI-anchor biosynthesis	sly00510	1	0.089	0.047	Cell surface protein modification

KEGG ID refers to the Kyoto Encyclopedia of Genes and Genomes pathway identifier. Hits indicate the number of high-confidence metabolites detected within a specific pathway. Raw *p* represents the nominal p-value calculated from the hypergeometric test, where values < 0.05 indicate statistical significance. Impact score is derived from pathway topology analysis based on out-degree centrality, reflecting the relative importance of the identified metabolites as network hubs.

### 3.11 Structural barrier reinforcement and very-long-chain fatty acids-mediated defense

**Figure 8:**
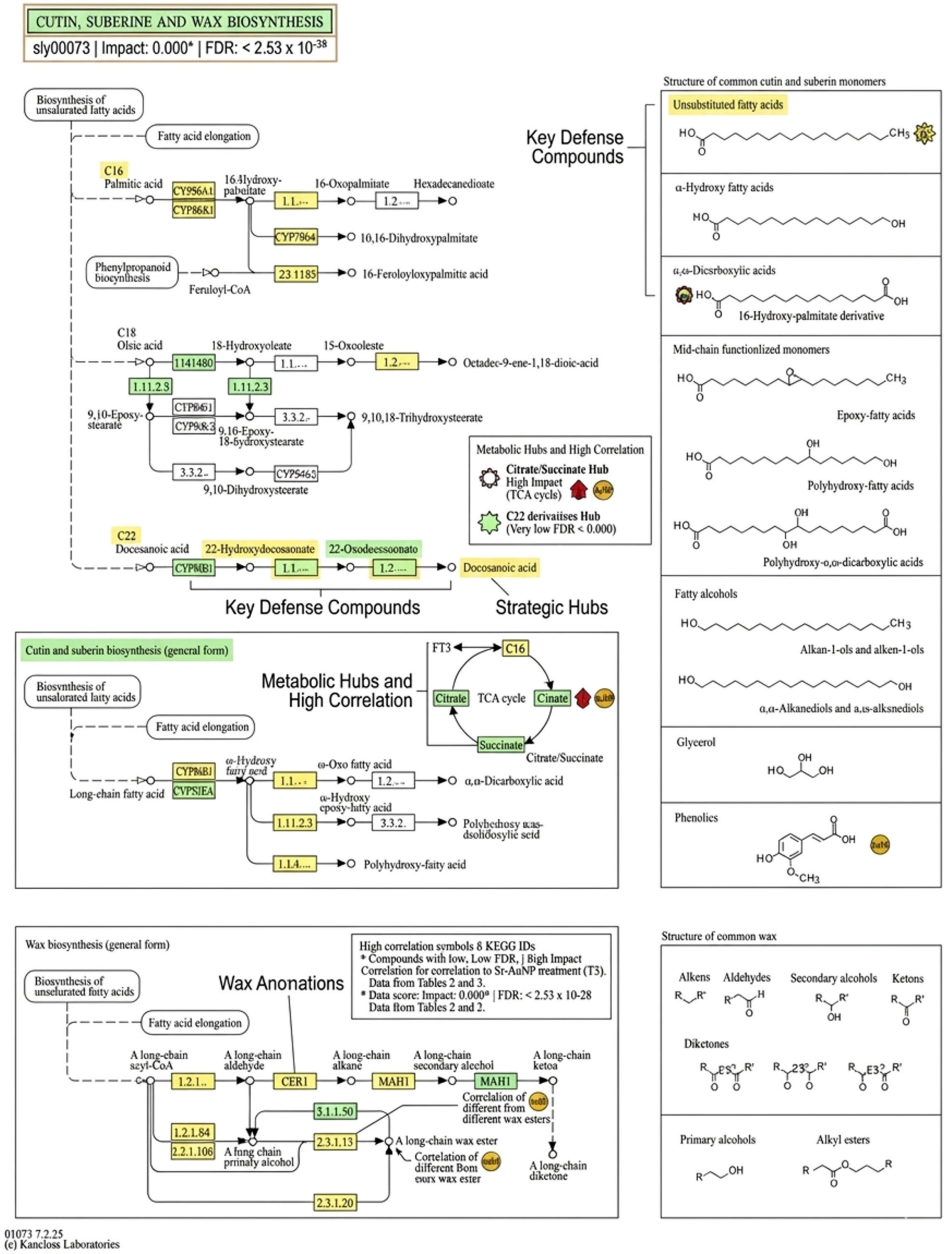
Schematic representation of the integrated Cutin, Suberin, and Wax biosynthesis pathways (adapted from KEGG map: sly00073). The metabolic network map illustrates the parallel biosynthetic routing of long-chain and very-long-chain fatty acids (VLCFAs) into specialized structural defense monomers. Highlighted is the metabolic utilization of the VLCFA biomarker Docosanoic acid (C22) via specialized enzymatic complexes (including CYP86A4, CYP86B1, and associated fatty acid elongases) to generate mid-chain functionalized monomers, α, ω-dicarboxylic acids, and fatty alcohols required for cell wall suberization and wax deposition. Strategic metabolic hubs and high-correlation indicators (including connections to the core citrate/succinate TCA cycle hub) trace the coordinated biochemical trade-offs sustaining root surface remodeling under *Erwinia persicina* challenge. Green and yellow highlighted nodes indicate targeted enzymatic steps involved in assembling these protective aliphatic polymers. Underlying pathway structures are adapted from Kanehisa Laboratories; reproduced with permission from KEGG.

To evaluate the structural modifications occurring at the host-pathogen interface, metabolic shifts pointing toward physical boundary reinforcement were investigated. The metabolic importance of these defense responses is emphasized by the simultaneous increase in activity of three different types of biosynthetic pathways, i.e., Cutin, Suberin and Wax biosynthesis (**Figure 8**). The metabolism of each of these pathways is centered around the production and elongation of Very-Long-Chain Fatty Acids (VLCFA; fatty acids having aliphatic chains ≥C20), which provide critical hydrophobic components of plant structural barriers.

Our metabolomic profiling identified a significant accumulation of the VLCFA precursor Docosanoic acid (C22), which acts as a fundamental hub driving these downstream protective networks. According to the reconstructed KEGG pathway architecture (**Figure 8**), Docosanoic acid undergoes sequential enzymatic functionalization via specialized cytochrome P450 monooxygenases (e.g., CYP86B1) and fatty acid omega (ω) hydroxylases. This leads to its conversion into ω-hydroxy fatty acids, α, ω -dicarboxylic acids, and corresponding fatty alcohols. These VLCFA-derived monomers are essential for the polymerization of the suberin lamellae matrix and the deposition of protective epicuticular wax layers. The targeted activation of these three independent lipid pathways suggests a strategic, multi-layered reinforcement of the tomato root’s physical barriers to arrest *Erwinia persicina* invasion. This defensive remodeling process is potentially primed and accelerated by the Sr-AuNPs conjugate (T3) treatment.

### 3.12 Functional biochemical connectivity and secondary metabolism networks

#### 3.12.1 Central carbon and energy metabolism

**Figure 9:**
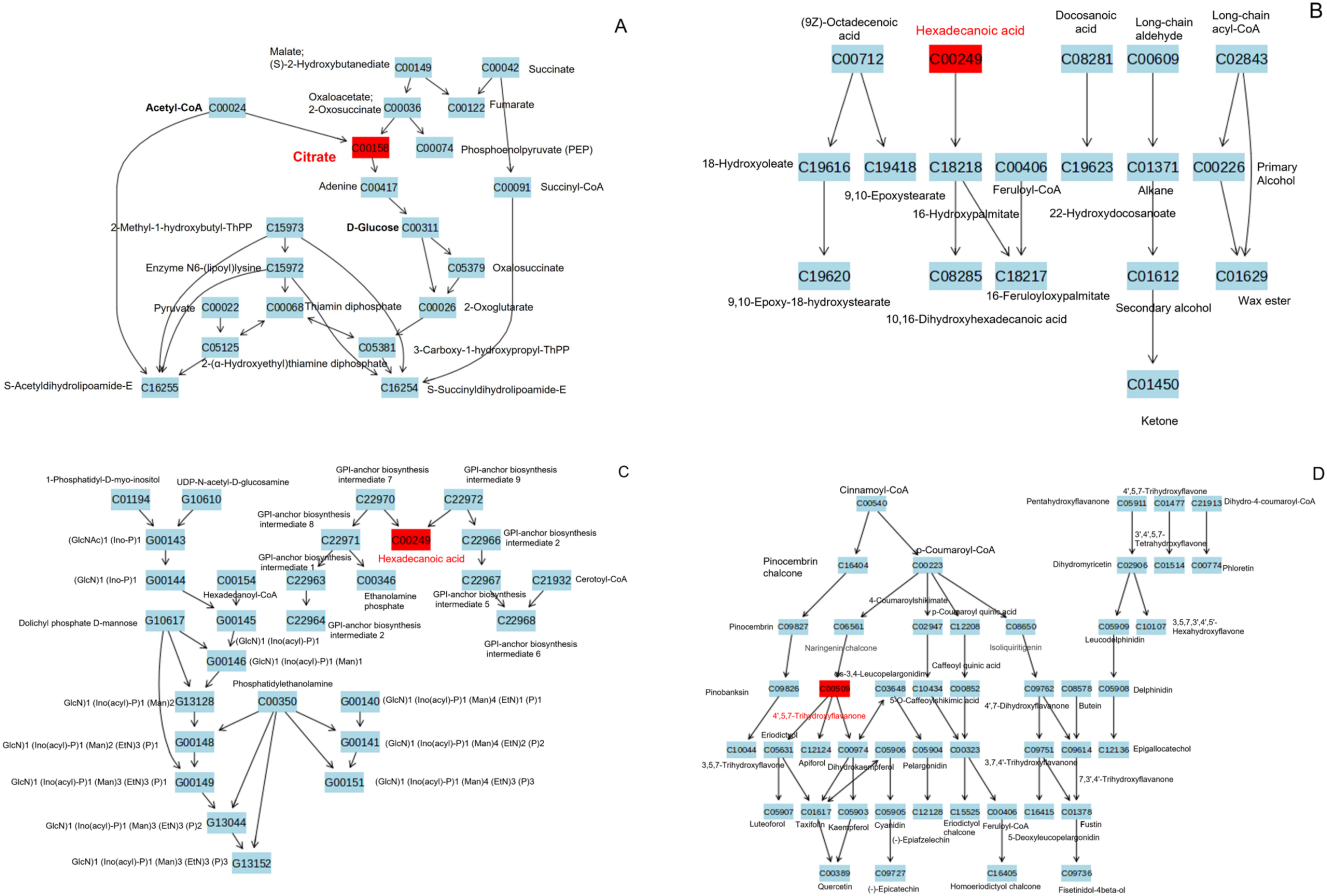
Integrated pathway topology and functional sub-network mapping of Sr-AUNP primed tomato roots. Global metabolic network reconstruction illustrates the multi-layered defense response of *Solanum lycopersicum* against *Erwinia persicina*. Analysis was performed using the MetaboAnalyst 6.0 Network Explorer tool mapped against the sly library. **(A)** Central Carbon Metabolism: Hub analysis of the TCA cycle identifying Citrate (C00158) as a primary energetic driver for defense. **(B)** Structural Reinforcement: Mapping of metabolic flux from Hexadecanoic acid (C00249) toward the biosynthesis of Wax esters (C01629). **(C)** Signaling Dynamics: Connectivity of lipid precursors in the assembly of GPI-anchor biosynthesis intermediates (G10617, G00143). **(D)** Secondary Defense: Network depicting the synthesis of antimicrobial flavonoids from Cinnamoyl-CoA (C00540) and p-Coumaroyl-CoA (C00223). Red nodes indicate high-impact pathway-associated features identified through MetaboAnalyst 6.0 (*p <* 0.05).

Topological analysis of the central carbon pathway network demonstrates that Citrate (C00158) functions as a primary metabolic hub (**Figure 9A**). Within the reconstructed Tricarboxylic acid (TCA) cycle, citrate serves as a critical junction point, mediating the condensation of input acetyl-CoA (C00024) and oxaloacetate (C00036). The structural pathway map reveals direct metabolic routes to D-glucose (C00311) biosynthesis and key energetic intermediates, including Succinate (C00042) and Malate (C00149). This highly connected network configuration underscores the metabolic capacity of the *S. rhizophila* strain to efficiently assimilate and transform diverse carbon substrates derived from tomato root exudates.

#### 3.12.2 Lipid metabolism and biofilm precursors

The study of lipid pathways shows that Hexadecanoic acid (C00249) can be used in many ways (**Figure 9B**). Hexadecanoic acid serves as a precursor for long-chain aliphatic compounds which include 16-HydroxyHexadecanoic acid (C08285) and various Wax esters (C01629). The hydrophobic derivatives function as essential components for constructing extracellular polymeric substances (EPS) which support biofilm development.

#### 3.12.3 Membrane and glycosylphosphatidylinositol -anchor assembly

The *Stenotrophomonas rhizophila* strain demonstrates the capacity to incorporate Hexadecanoic acid into complex membrane components (**Figure 9C**). The metabolic flow produces both Phosphatidylethanolamine (C00350) and Glycosylphosphatidylinositol (GPI) intermediates which include G00146 and G13128 and G13152. This capability for sophisticated membrane remodeling likely facilitates stable root colonization and stress resistance.

#### 3.12.4 Secondary metabolism: flavonoid processing

The metabolic profile includes an expansive Flavonoid biosynthesis pathway (**Figure 9D**). The strain possesses enzymatic architecture to process Cinnamoyl-CoA (C00540) and p-Coumaroyl-CoA (C00223) into a wide array of Flavonoids which includes Naringenin (C00509). The downstream metabolic pathway produces powerful antioxidant compounds together with signaling molecules which include Kaempferol (C05903) and Quercetin (C00389) and Epicatechin (C09727). Metabolites function as essential components which enable plants to communicate with microbes while plants defend against oxidative damage that occurs during pathogen attacks.

The sub-network reconstruction via Network Explorer tool (**Figures 9A–D**) demonstrated in better detail, the interconnection between primary and secondary metabolism. Clearly, metabolic flux can be seen directed from the main nexus Hexadecanoic acid (C00249) towards the synthesis of Wax esters (C01629) and components needed to build GPI anchors (G10617, G00143). Citrate (C00158) can also be defined as a ‘Regulatory Pivot’ connecting the TCA cycle to Flavonoid biosynthesis (sly00941; Impact: 0.103), part of the antimicrobial pathway.

## 4 Discussion

This work provides evidence at multi-level to prove successful conjugation of phycosynthesized AuNPs with *Stenotrophomonas rhizophila*. Surface Plasmon Resonance (SPR) peak at ~534 nm corresponds to formation of nanosized gold particles and stable colloidal system maintained by algal metabolites as reducing and capping agents (Ramakrishna et al., 2016). TEM indicates direct association and not coexistence of AuNPs on bacterial surfaces. The dense distribution of nanoparticles can be attributed to high affinity between AuNPs and the cell wall constituents of *Stenotrophomonas rhizophila*. Initially, extracellular polymeric substances (EPS) mediate nanoparticle adhesion, followed by the subsequent stability of the metal clusters (Fulaz et al., 2019; Joshi et al., 2020). Enhancement of hydrodynamic size along with intermediate zeta potential confirms formation of a stable nano-bio-hybrid interface. Decreased negative charge in the hybridized system can be assigned to electrostatic attraction with AuNPs which causes some extent of charge neutralization (Dong et al., 2018; Pajerski et al., 2019).

FTIR spectral changes provide compelling evidence of conjugation occurring at the molecular level. The roles of amide I and II, along with carbohydrate groups, indicate the specific functional and chemical groups involved in the interaction. These groups act as effective binding sites on the bacterial surface proteins and polysaccharides leading to the immobilization of the nanoparticles (Yusoff et al., 2022). This interaction of metal nanoparticles with cell envelope follows the same route of ‘bio-organic corona’ formation with proteins/polysaccharides acting as a support.

ICP-MS analysis proves the quantitative binding of AuNPs on the hybridized system as the gold concentration is significantly high in the conjugated samples which correlate with observed structural and spectroscopic changes (Allabashi et al., 2009; Kuznetsova et al., 2020). The above results prove that conjugation is electrostatics and/or bio-molecular in nature and it creates a stable bio-nano system that promotes the bacterial efficiency especially the plant-microbe interactions in plant rhizosphere by enhancing root colonization, and the modulation of bacterial physiological functions through nanoparticle action.

Conjugation of PGPR strains through the nanoparticle scaffold constitutes an important pre-requisite in initiating the metabolic reprogramming process as shown in **Table 3**. Increased adhesion capacity ensures enhanced activation of signaling pathways involved in ISR. This has been shown by high topology impact of the TCA cycle (0.115) as well as Cutin and Suberin biosynthesis (P-value = 0.0466).

This study demonstrates that rhizobacterial priming and nanoparticle-conjugated rhizobacterial treatments are associated with distinct metabolic states in tomato roots during *E. persicina* challenge. The detection of a C22 fatty acid derivative (m/z 338.33) as a primary biomarker in pathogen-treated roots highlights a systemic shift in the root lipidome during infection (**Figure 5**). These Very-Long-Chain Fatty Acids (VLCFAs) are essential components of plant defense. They serve as precursors for protective surface barriers and as integral constituents of the plasma membrane that modulate defense signaling (Batsale et al., 2021).

The significant accumulation of the C22 fatty acid derivative suggests that the plant undergoes active lipid remodeling to counteract biotic stress. Such modifications to the fatty acid profile are known to facilitate lipid-mediated signaling pathways, which are critical for the activation of downstream host defense responses (Hou et al., 2016; Kuźniak and Gajewska, 2024). Consequently, the strong contribution of the C22 derivative to group separation in our models identifies it as a robust metabolic signature of a pathogen-responsive state. This shift underscores the plant’s strategy of mobilizing long-chain lipid biosynthetic pathways to enhance physiological resilience against invading pathogens (Wang et al., 2025; Jha et al., 2025).

By disarming the pathogen *Erwinia persicina* instead of killing it, our antivirulence mechanism is likely under lower pressure to develop resistance while still providing protection for the host plant. This mechanism of action classifies erucamide as a functional survival effector, which lies at the intersection of metabolic reprogramming and immune responses. The observed tissue accumulation of erucamide in response to pathogens indicates an adaptive evolution towards chemical defense strategies that are likely to complement physical barrier-based defense mechanisms.

In plants treated with *S. rhizophila* alone, the increased relative abundance of Benzoic acid suggests engagement of phenylpropanoid-related metabolism in a primed but metabolically stable condition. This agrees with previous studies showing that ISR often involves limited basal metabolic perturbation prior to pathogen attack (Moore et al., 2002; Kumawat et al., 2019; Singh., 2025).

The AuNP-conjugated rhizobacterial treatment was associated with coordinated modulation of central carbon metabolism and secondary metabolic pathways. The reduction of several primary metabolite signals alongside increased importance of lipid- and phenolic-associated features indicates redistribution of metabolic investment under stress (Frei et al., 2018; Panichikkal et al., 2019). In this study, metabolic drag refers specifically to the observed reduction in relative abundance of primary metabolite signals and does not represent a direct measurement of growth– defense trade-offs.

### 4.1 Pathway topology and metabolic coordination of Sr-AuNP stimulation

Metabolomic profiling analysis via pathway topology indicates that there exists a considerable restructuring of metabolic networks within the tomato root tissue when subjected to the priming process by Sr-AuNP. The data show that the plant shifts from the metabolic burden due to the *E. persicina* infection to the coordination of defensive mechanisms by activating both the primary energy metabolism and structural reinforcement pathways.

### 4.2 Defensive structural reinforcement and role of lipid amides

The first important effect induced by the stimulation of ISR by Sr-AuNP is the reinforcement of the cell wall structure within the roots. As indicated by pathway enrichment analysis, the activation of the Cutin, Suberin, and Wax biosynthesis pathway (sly00073; p = 0.0466) is evident (**Table 3**). It is important to note that the role of lipid amides in creating the defensive structure of plants was experimentally proven (Chen et al., 2022; El-Gazzar et al., 2025). Lipid amides constitute an important component of Suberin and Cutin aliphatic domains and provide physical protection against pathogens (Philippe et al., 2020; Vishwanath et al., 2015).

The upregulation of the pathway further supports the idea of the “structural priming” action of Sr-AuNP conjugates, as indicated by the modulations of Glycerolipid metabolism (sly00561) and Glycosylphosphatidylinositol-GPI-anchor biosynthesis (sly00510). These processes are crucial for structural alterations, membrane reorganization, and protein anchoring on the plasma membrane of defense signaling components (Tamilmani et al., 2016; Yeats et al., 2018).

### 4.3 Energy allocation and TCA cycle hub

To meet the energy demands associated with these changes in the structural composition, according to the pathway topology analysis, Citrate cycle (TCA cycle) (sly00020; Impact: 0.115) and Flavonoid biosynthesis (sly00941; Impact: 0.103) were the major regulatory hubs affected. Considering the role of TCA cycle in carbon allocation and precursor production, and Flavonoid action in redox regulation and signaling, the simultaneous alteration of the two pathways is reasonable (Yang et al., 2025; Feng et al., 2026; Akhtar et al., 2026).

The high topology effect of the TCA cycle, particularly the Citrate cycle, indicates that plants treated with Sr-AuNP exhibit a strong carbon flux that acts as a supplier of systemic resistance (Bolton, 2009; Serag et al., 2023). The sustained carbon flux ensures sufficient energy (ATP) and precursors required to synthesize antibacterial phenolics through the Flavonoid’s pathway and ROS detoxification (Davies et al., 2024; Zheng et al., 2024). In addition, the modulation of Glyoxylate and Dicarboxylate metabolism (sly00630) implies an improved efficiency of carbon recycling under stress conditions (Nunes-Nesi et al., 2016).

The notable upregulation of the Cutin and Suberin biosynthesis pathway (*p <* 0.046) is experimental evidence for the involvement of lipid amides, such as 13-docosenamide, in building the defense system of the plant (Chen et al., 2022). This indicates that the primary focus of Sr-AuNP stimulation in ISR resistance to *Erwinia persicina* infection lies in building the cell wall barrier in roots. However, because pathway inference is based on a limited number of detected metabolites, these results identify candidate regulatory nodes within the measured network rather than direct changes in pathway flux.

The concept of metabolic drag can be interpreted within the broader framework of the well-established growth–defense trade-off in plants. However, it should be noted that this mechanism has not directly validated the present study. Extensive experimental evidence across plant systems demonstrates that activation of immune responses imposes a metabolic cost, leading to reduced growth and biomass accumulation. This trade-off arises from the allocation of limited resources toward defense-related processes, including the synthesis of antimicrobial compounds, phenolics, and signaling molecules (Vega-Álvarez et al., 2023). Recent studies further indicate that pathogen or herbivore attack triggers metabolic reprogramming through phytohormonal crosstalk, prioritizing defense over, growth processes (Frungillo,2023). Such responses are often accompanied by decreased primary metabolism and altered energy distribution, reflecting a shift in resource allocation toward protective functions.

Although these results offer a revealing insight into nano-primed resistance, it is important to recognize that pathway inference was performed using only a selected subset of high-confidence metabolites. Therefore, the results presented herein represent the identified potential regulatory nodes of the observed network, rather than direct evidence of pathway flux alterations. Further investigations using ^13C isotopic labeling, gas exchange measurements, and expanded multi-omics analysis will be required to provide complete metabolic coverage. Nonetheless, our study has identified a clear metabolic signature that the success of Sr-AuNP priming arises from the coordination of central energy production with reinforcement of the cellular wall in *S. lycopersicum*.

FTIR spectral shifts further support treatment-dependent redistribution between carbohydrate- and lipid-associated biochemical components, indicating structural and metabolic reconfiguration of root exudates. These alterations are in line with the concept of adaptive remodeling in the biochemical makeup of the plant in response to biotic and nano-enabled stresses (Nikalje et al., 2019; Bozkurt-Girit and Kilic, 2025). However, it is noteworthy to mention that the study did not directly quantify “metabolic drag” instead, the analysis was based on the alterations in the pathways and the relative levels of the metabolites. Consequently, the reported trends reflect shifts in metabolism consistent with established growth-defense trade-offs. To elucidate the mechanistic basis of the responses more conclusively, these findings should be validated through integrative multi-omics approaches.

The study has established a framework in understanding the nano-enabled rhizobacterial priming based on metabolomics. Additionally, the study has demonstrated the potential benefits associated with the integration of metabolomics with transcriptomics, enzymology, and the physiological approaches in understanding the functional consequences associated with the bio-nano composite-mediated stress.

## 5 Conclusion

This study provides an integrated, multi-platform metabolomic characterization of tomato root exudate responses to *Erwinia persicina* under rhizobacterial and nanoparticle-assisted priming. The results demonstrate that nanoparticle-conjugated *Stenotrophomonas rhizophila* is associated with a distinct metabolic configuration during pathogen challenges. Specifically, univariate analysis identified seven high-confidence metabolic markers (q < 0.05) involving significant shifts in organic acids, lipid-like amides, and aromatic derivatives. Based on pathway topology analysis, the TCA cycle and Flavonoid biosynthesis emerge as putative coordination points. However, given the high false discovery rates (q > 0.85) and low feature hits (1 per pathway), these results are considered preliminary and associated rather than definitive evidence of metabolic flux. The current data suggests a rearrangement of primary and secondary metabolic profiles, but the actual kinetic rates of these processes remain unquantified.

A significant limitation of this study is the absence of an AuNP-only control, which prevents the complete disentanglement of independent nanoparticle effects from those of the bacterial inoculant. Furthermore, the lack of direct phenotypic data, such as disease severity or plant biomass, means that functional resistance cannot yet be confirmed. We conclude that while nanoparticle-assisted rhizobacterial priming is associated with a distinct metabolic state, causal mechanisms regarding regulatory dominance or “metabolic drag” remain speculative. Future studies integrating untargeted metabolomics, fluxomics, and transcriptomic validation will be essential to confirm the functional significance and regulatory dominance of the pathways identified in this preliminary analysis.

## Data Availability

The original contributions presented in the study are included in the article/**Supplementary Material**. Further inquiries can be directed to the corresponding author.
