# Supplementary material for: Metabolic reprogramming of tomato roots during rhizobacteria-mediated defense against Erwinia persicina: modulation by gold nanoparticle conjugation
Source: Front Plant Sci. 2026 Jun 17;17:1824882. doi: 10.3389/fpls.2026.1824882 (PMC13319062; doi:10.3389/fpls.2026.1824882)
Supplement: Supplementary file 1 [file Table1.docx]

**Supplementary Table S1**

**Table S3. Chemical Annotation and Database Mapping of Key Discriminant Metabolites in the Tomato Rhizosphere.**

| **Metabolite** | **KEGG ID** | **HMDB ID** | **SMILES String** |
| --- | --- | --- | --- |
| Benzoic acid | C00180 | HMDB0001870 | OC(=O)C1=CC=CC=C1 |
| Citric acid | C00158 | HMDB0000094 | OC(=O)CC(O)(CC(O)=O)C(O)=O |
| L-Valine | C00183 | HMDB0000883 | CC(C)[C@H](N)C(O)=O |
| Naringenin | C00509 | HMDB0002670 | OC1=CC=C(C=C1)[C@@H]1CC(=O)C2=C(O1)C=C(O)C=C2O |
| Palmitic acid | C00249 | HMDB0000220 | CCCCCCCCCCCCCCCC(O)=O |

**Note:** This table provides standardized chemical identifiers, including KEGG ID, HMDB ID, and SMILES strings, used to verify the biological identity of the primary metabolic hubs identified across the integrated LC-MS/MS, GC-MS, and ^1H NMR datasets.
